# A Dual‐Cation Exchange Membrane Electrolyzer for Continuous H_2_ Production from Seawater

**DOI:** 10.1002/advs.202401702

**Published:** 2024-04-03

**Authors:** Yongwen Ren, Faying Fan, Yaojian Zhang, Lin Chen, Zhe Wang, Jiedong Li, Jingwen Zhao, Bo Tang, Guanglei Cui

**Affiliations:** ^1^ Qingdao Industrial Energy Storage Research Institute Qingdao Institute of Bioenergy and Bioprocess Technology Chinese Academy of Sciences Qingdao 266101 China; ^2^ Shandong Energy Institute Qingdao 266101 China; ^3^ Qingdao New Energy Shandong Laboratory Qingdao 266101 China; ^4^ Laoshan Laboratory Qingdao 266237 China; ^5^ School of Future Technology University of Chinese Academy of Sciences Beijing 100049 China

**Keywords:** electrocatalysis, green hydrogen production, seawater splitting, water migration balance

## Abstract

Direct seawater splitting (DSS) offers an aspirational route toward green hydrogen (H_2_) production but remains challenging when operating in a practically continuous manner, mainly due to the difficulty in establishing the water supply–consumption balance under the interference from impurity ions. A DSS system is reported for continuous ampere‐level H_2_ production by coupling a dual‐cation exchange membrane (CEM) three‐compartment architecture with a circulatory electrolyte design. Monovalent‐selective CEMs decouple the transmembrane water migration from interferences of Mg^2+^, Ca^2+^, and Cl^−^ ions while maintaining ionic neutrality during electrolysis; the self‐loop concentrated alkaline electrolyte ensures the constant gradient of water chemical potential, allowing a specific water supply–consumption balance relationship in a seawater–electrolyte–H_2_ sequence to be built among an expanded current range. Even paired with commercialized Ni foams, this electrolyzer (model size: 2 × 2 cm^2^) continuously produces H_2_ from flowing seawater with a rate of 7.5 mL min^−1^ at an industrially relevant current of 1.0 A over 100 h. More importantly, the energy consumption can be further reduced by coupling more efficient NiMo/NiFe foams (≈6.2 kWh Nm^−3^ H_2_ at 1.0 A), demonstrating the potential to further optimize the continuous DSS electrolyzer for practical applications.

## Graphical abstract

A dual‐cation exchange membrane three‐compartment architecture with a circulatory electrolyte design is developed for green H_2_ production directly from natural seawater. Based on an established water transport balance, this electrolyzer can continuously produce H_2_ over 100 h at an industry‐relevant current of 1.0 A, while avoiding the precipitate formation and chlorine corrosion issues.

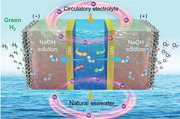

## 1 Introduction

**Figure 1:**
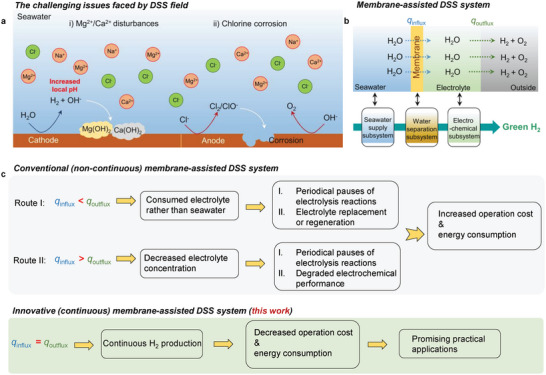
a) Illustration for the fundamental issues faced by DSS for H_2_ production. b) Scheme of the emerging membrane‐assisted DSS system, primarily composed by a seawater supply subsystem, a water separation system (typically based on membrane separation processes), and an electrochemical subsystem, to circumvent the issues caused by impurity ions. c) Comparison of the conventional non‐continuous and the proposed continuous DSS systems, particularly involving the water supply–consumption balance.

Green hydrogen (H_2_), generated by using sustainable electricity to split water, is capable of driving vehicles and decarbonizing industries, but its scalable production also exacerbates global freshwater shortages.^[^ ^1^, ^2^ ^]^ Direct seawater splitting (DSS) without using chemical additives (buffers or bases) and complex desalination processes has been actively pursued as an appealing pathway for green H_2_ production.^[^ ^3^, ^4^, ^5^, ^6^ ^]^ As an emerging field, the development of this technique is still in its infancy and retarded by several fundamental issues, typically including the disturbances of unwanted ions (e.g., Mg^2+^, Ca^2+^ cations) and the corrosion of chlorine species, finally leading to relatively low energy efficiency and poor stability (**Figure** **1**a).^[^ ^7^, ^8^, ^9^, ^10^ ^]^ In particular, Mg^2+^ (≈1300 ppm) and Ca^2+^ (≈400 ppm) cations in seawater can form precipitations over the cathodes due to the locally increased pH values during the H_2_ evolution reaction (HER), blocking the charge transfer and thereby degrading the whole reaction.^[^ ^11^, ^12^, ^13^ ^]^ Moreover, Cl^−^ ions (≈20000 ppm in seawater) can also be easily oxidized into ClO^−^ ions (Cl^−^ + OH^−^ → ClO^−^ + H_2_O + 2e^−^, pH > 7.5), which severely competes with the expected oxygen evolution reaction (OER) and corrodes the anode components.^[^ ^14^, ^15^, ^16^, ^17^ ^]^

Conventionally, although the interferences from harmful Cl^−^, Mg^2+^, and Ca^2+^ ions (seawater) can be mitigated by engineering catalysts with tailored microstructures such as introducing Lewis acid layers, the structural durability remains to be concerned in view of the elusive and mutable local microenvironments over catalysts under operando conditions (e.g., the fluctuation of local pH).^[^ ^18^, ^19^, ^20^, ^21^, ^22^ ^]^ Alternatively, coupling electrolyzers with membrane technologies (e.g., using semipermeable membranes) provides a more effective tactic to selectively pre‐extract water from seawater (Figure 1b), but most of reported integrated systems suffer from large ohmic resistances and the remaining chlorine corrosion issue.^[^ ^23^, ^24^, ^25^ ^]^ Very recently, a “liquid–gas–liquid” water extraction manner along with resistance to impurity ions was reported by using porous waterproof membranes; its deployment could be constrained by the limited water supply (involving multistep phase transitions) rates.^[^ ^26^ ^]^ Also, it was revealed that the Na^+^ ion exchange membrane can effectively inhibit the Cl^−^ migration from seawater to electrolyte; however, the practical application of the corresponding electrolyzer in natural seawater was still limited due to the unresolved precipitation issues of Mg^2+^ and Ca^2+^ ions at the cathode side.^[^ ^27^ ^]^

From a cost‐effective viewpoint, the ideal DSS process should be operated continuously without the needs for periodical pauses of electrolysis reactions and replacement/regeneration of electrolytes (denoted as continuous DSS). This target puts forward an additional requirement of formulating a water supply–consumption balance—the water migration rate equaling the water consumption rate, *q*_influx_ = *q*_outflux_ (Figure 1b,c).^[^ ^11^, ^28^, ^29^, ^30^ ^]^ Unfortunately, although few reports touched the topic of the water balance, continuous DSS operation with long‐term stability has not been identified, especially under practically feasible currents, primarily because of the lack of a robust and constant driving force for fast transmembrane water transport under interferences from harmful ions in seawater.

In this study, continuous DSS for ampere‐level H_2_ production is successfully accomplished with a combination of a dual‐cation exchange membrane (CEM) three‐compartment configuration and a circulatory electrolyte design (Figure 1c). The monovalent‐selective CEMs spatially separate the seawater (flowing feed) chamber from bilateral electrolyte (concentrated NaOH) chambers, ensuring the sustainable water supply from seawater without interferences from Mg^2+^, Ca^2+^, and Cl^−^ ions as well as the ionic neutrality by permselective monovalent cation carriers (e.g., Na^+^ ions). Moreover, circulating the NaOH electrolyte eliminates both concentration and pH differences between cathode and anode sides during operation, which maintains a constant transmembrane concentration gradient (Δ*C*), namely, the driving force for water migration. As such, a specific relation of the water transport balance can be formulated among an expanded current range (beyond 1.0 A) according to Fick's law and Faraday's law. Such a dual‐CEM continuous DSS system equipped with commercial Ni foams can ran at an industry‐relevant current of 1.0 A over 100 h with a H_2_ production rate of 7.5 mL min^−1^. The energy consumption of the system can be further reduced by upgrading the catalysts, highlighting the foreseeable practical applications. This electrolyzer design provides an alternative solution to the problems inherent in green H_2_ production from low‐grade/impure water feeds.

## 2 Results and Discussion

### 2.1 Overview of the Continuous DSS System

**Figure 2:**
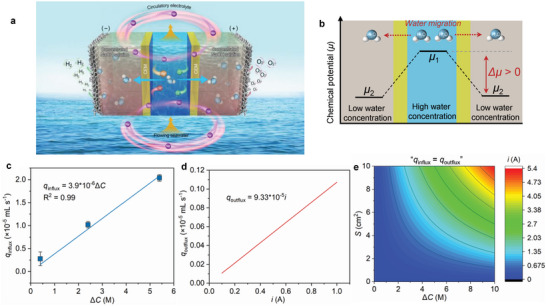
a) Schematic of the continuous DSS system, based on a dual‐CEM three‐compartment architecture integrated with a circulatory electrolyte design. HER and OER take place at cathode and anode in the circulating NaOH electrolyte, respectively. b) Mechanism of water migration from the central seawater chamber to the two‐sided electrolyte chambers across the CEMs based on the second law of thermodynamics. c) Experimentally determined relationship between *q*_influx_ of CEM (Gore) and Δ*C*. d) Theoretical relationship between *q*_outflux_ and *i* calculated according to Faraday's law. Note that the relationship is established assuming that both the Faradaic efficiencies (FEs) of HER and OER are 100%. e) Heatmap of the predicted *i* as a function of Δ*C* (*x* axis) and *S* (*y* axis) in the continuous DSS system.

The proposed continuous DSS system for green H_2_ production consists of a seawater chamber in the middle and two electrolyte (concentrated NaOH solution) chambers on cathode and anode sides, respectively (**Figure** **2**a). Three chambers are spatially and physically separated by two monovalent‐selective CEMs that maintain the cross‐chamber ionic neutrality due to the directed diffusion of monovalent cations (contributed mainly by Na^+^ ions given its dominant presence in the whole system) under the applied electric fields.^[^ ^31^, ^32^ ^]^ Meanwhile, the disturbing ions, e.g., Mg^2+^, Ca^2+^, and Cl^−^ ions, can be effectively intercepted due to the unique design of ion transport channels in the monovalent‐selective CEMs.^[^ ^33^, ^34^ **^]^** Notably, the NaOH electrolytes in cathode and anode chambers are continuously circulated with external pumps, eliminating the differences in ionic concentrations and pH values between cathode and anode sides during electrolysis.

To enable the water migration from seawater to electrolyte, the NaOH solutions with concentrations higher than 0.6 m are selected as the electrolyte given the typical salinity of ≈3.5 wt% (corresponding to ≈0.6 m of NaCl concentration) in seawater.^[^ ^24^ ^]^ According to the second law of thermodynamics, the chemical potentials of water in seawater (*μ*_1_) and electrolyte (*μ*_2_) can be expressed as follows.

(1) $$\mu_{1}=\mu_{0}+RT\ln\frac{\gamma c_{1}}{c^{*}}$$

(2) $$\mu_{2}=\mu_{0}+RT\ln\frac{\gamma c_{2}}{c^{*}}$$

where *μ*_0_ is the chemical potential of water in the standard state, *R* is the gas constant (8.314 J mol^−1^ K^−1^), *T* is the thermodynamic temperature (K), *c*_1_ and *c*_2_ are the concentrations of water in seawater and electrolyte, respectively, *γ* is the activity coefficient of water, and c* is equal to 1 mol L^−1^. Thus, the difference of the chemical potential of water between seawater and electrolyte (Δ*μ*) can be described as follows.

(3) $$\Delta \mu =\mu_{1}-\mu_{2}=RT\ln\frac{c_{1}}{c_{2}}$$

Given the lower salt concentration of seawater compared with that of the adopted electrolyte, the concentration of water in seawater is higher than that in the electrolyte (*c*_1_ > *c*_2_), that is, the Δ*μ* value is greater than zero (Figure 2b). Under this driving force, water molecules in seawater have a strong tendency to migrate from seawater to electrolyte through CEMs. A combination of the flowing seawater feeding and the electrolyte circulation maintains the constancy of the Δ*μ* value for stabilizing transmembrane water migration rates during continuous DSS.

### 2.2 Water Balance Management

To establish a relationship of the water dynamic balance for a continuous DSS system (*q*_influx_ = *q*_outflux_), we took a commercial GORE‐SELECT Gore M788.12 (donated as Gore, see Table S1, Supporting Information for details) CEM as an example and systematically studied the transmembrane water migrate rate. According to Fick's law, *q*_influx_ (mL s^−1^) across membranes with a known surface area (*S*, cm^2^) is proportional to Δ*C* (M) between seawater and electrolyte, as described as follows.^[^ ^35^ ^]^

(4) $$q_{\mathrm{influx}}=DS\Delta C$$

where *D* is the permeability coefficient of membranes, which is related to the intrinsic properties of the membranes (e.g., membrane thickness) and can be experimentally determined (see Supporting Information for details). As expected, the measured *q*_influx_ gradually increases with the increase of Δ*C* (Figure S1, Supporting Information); further, the fitting curve of *q*_influx_ and Δ*C* exhibits a good linear relationship with an equation of *q*_influx_ = 3.9 × 10^−6^Δ*C* (*R* ^2^ = 0.99) (Figure 2c). *D* can be calculated to be 5.0 × 10^−6^ based on the known surface area of CEM (0.785 cm^2^). Thus, *q*_influx_ from seawater to electrolyte through CEMs can be specifically described as follows.

(5) $$q_{\mathrm{influx}}=\;5.0\times 10^{-6}S\Delta C$$

*q*_outflux_ is related to the current (*i*, A) consumed during the electrolysis process following Faraday's law,^[^ ^23^ ^]^

(6) $$q_{\mathrm{outflux}}=\frac{iV_{m}}{2F}=\;9.33\;\times \;10^{-5}i$$

where *V*_m_ and *F* are the molar volume of H_2_O (18 mL mol^‐1^) and the Faraday constant (96485.3 C mol^−1^), respectively. It is revealed that *q*_outflux_ is linear with *i* (Figure 2d). Equating Equations (5) and (6), the conditions for a continuous DSS system (*q*_influx_ = *q*_outflux_) involving *i* and Δ*C* can be described as follows.

(7) i=0.054SΔC

To better understand this point, the relation between *i*, *S*, and Δ*C* is further presented in Figure 2e. It is apparent that *i* is positively related to *S* and Δ*C*. This relationship allows us to realize a practically viable continuous DSS system (with known *S*) for green H_2_ production at a given *i* value by regulating Δ*C*, where the water dynamic balance is ensured.

### 2.3 Continuous DSS Demonstration with Simulated Seawater

**Figure 3:**
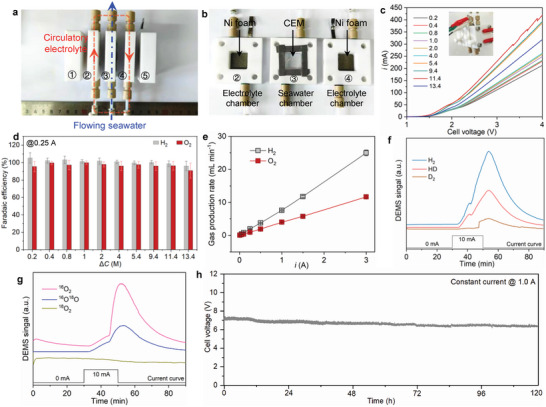
a,b) Digital photos for the disassembled units of our DSS electrolyzer. c) LSV curves of DSS with various Δ*C* ranging from 0.2 to 13.4 m. Inset is the digital photo of the assembled electrolyzer. d) FEs of HER and OER of the DSS system with different Δ*C* performed at a constant current of 0.25 A. e) Gas production rates of the system conducted at different currents for 1 h. f,g) DEMS signals of H_2_, HD, and D_2_ f) as well as ^16^O_2_, ^16^O^18^O, and ^16^O_2_ g) collected from the cathode (using D_2_O as the solvent of the simulated seawater) and anode sides (using H_2_^18^O as the solvent of the simulated seawater), respectively, during electrolysis being enabled by a constant current of 10.0 mA. h) Galvanostatic electrolysis curve of continuous DSS performed in simulated seawater and a circulating 2.9 m NaOH electrolyte.

To verify the validity of the above relationship, a simulated seawater solution with a certain ionic concentration (e.g., 0.6 m NaCl) was first introduced into our dual‐CEM electrolyzer. The as‐made electrolyzer is presented in **Figure** **3**a,b, in which both the sizes of Gore CEMs and commercial Ni foams (Figure S2, Supporting Information, note that all electrode materials used in this work are Ni foams) were fixed at 2 × 2 cm^2^ (see Figure S3, Supporting Information, for whole system). The key feature of this electrolyzer consists in the design combining flowing seawater (or simulated seawater) with a circulatory NaOH electrolyte. The linear sweep voltammetry (LSV) curves performed in the system vary significantly at various Δ*C* values from 0.2 to 13.4 m (based on varying the concentration of the NaOH electrolyte), indicative of the critical role of Δ*C* in affecting seawater splitting performance (Figure 3c). The results derived from LSV curves (Figure S4a, Supporting Information) obviously reveal an initial increase of current as a function of Δ*C* followed by a drastic reduction when Δ*C* is greater than 11.4 m. This performance trend of electrolysis could be attributed to the difference in the conductivity of NaOH solutions, as reflected by the result that the smallest ohmic drop is also obtained at Δ*C* of 11.4 M (Figure S4b, Supporting Information).

In the whole range of Δ*C* from 0.2 to 13.4 m, both FEs of HER and OER maintain nearly 100% at a current of 0.25 A (Figure 3d). Of note, the near‐unity FE for OER suggests that the intractable issue of chlorine corrosion can be resolved, which is a reasonable phenomenon given the cationic selectivity of CEMs. Attractively, alkaline electrolysis conditions also endow the electrolyzer with inherently robust resistance against the chlorine evolution reaction, although the permeation tests show that a certain amount of Cl**^−^** ions can pass through the CEM after 96 h (Figure S5, Supporting Information).^[^ ^14^, ^36^ ^]^ To further verify this point, we then determined the possible chlorine products (HClO or ClO**^−^**) of chlorine evolution reaction in the anolyte by a modified DPD method (Figure S6, Supporting Information).^[^ ^37^, ^38^ ^]^ The results show that no HClO/ClO**^−^** products can be detected after electrolysis at 0.25 A for 1 h in the whole range of Δ*C* from 0.2 to 13.4 m (Figure S7, Supporting Information). We also investigated the DSS performance at ampere‐level operation currents. In an expanded current window up to 3.0 A, the system delivers exclusive selectivity for HER and OER with both FEs of approximately 100% (Figure S8, Supporting Information). The corresponding H_2_/O_2_ production rates almost linearly increase as the current augments, reaching 25.0 (±0.9) and 11.7 (±0.3) mL min**^−^** ^1^ at 3.0 A for H_2_ and O_2_, respectively (Figure 3e).

To investigate the possible active sites of Ni foams for HER and OER, we examined the electrochemical process with in situ Raman spectroscopy. No obvious changes can be observed in the structure of Ni foam during HER under different applied potentials (Figure S9, Supporting Information), indicative of Ni^0^ as the active species for HER. In comparison, two Raman peaks appear at the potential of 1.4 V during OER (445 cm^−1^: Ni─O(H) stretching vibration of β‐Ni(OH)_2_; 520 cm^−1^: Ni─O stretching vibration of β‐Ni(OH)_2_), whose intensity significantly enhances with the increase of applied potential.^[^ ^39^, ^40^ ^]^ This manifests the in situ constructed β‐Ni(OH)_2_ species over the Ni foams is responsible for OER, which is consistent with the previous reports.^[^ ^41^ ^]^ Also, to pursue the origin of water for produced H_2_ and O_2_, we then conducted isotope labeling experiments using D_2_O and H_2_^18^O as the solvents of simulated seawater, respectively, wherein the gas compositions generated by the system were analyzed with differential electrochemical mass spectrometry (DEMS) (Figure S10, Supporting Information).^[^ ^42^ ^]^ When using D_2_O for simulated seawater, the signals of HD and D_2_ were both observed from the cathode gas products after electrolysis at 10 mA for 20 min (Figure 3f), indicating that D_2_O in seawater chamber can pass through CEM and diffuse into the cathode chamber. This is also the case for H_2_^18^O‐contained simulated seawater, as proved by the detected signal of ^16^O^18^O apart from ^16^O_2_ after electrolysis (Figure 3g). These results prove the feasibility of using Δ*C* as the driving force for transmembrane water migration despite abundant Cl^−^ ions being present, which is the prerequisite for building the water dynamic balance for continuous DSS.

Given the size of the adopted CEMs (2 × 2 cm^2^, corresponding to *S* = 8 cm^2^), the theoretical relationship between *i* and Δ*C* can be further formulated as *i* = 0.43Δ*C*. To acquire a practically usable current of 1.0 A for continuous DSS, we thus employed a Δ*C* value of 2.3 m (corresponding to a 2.9 m NaOH electrolyte) as the driving force for water migration. The constructed system can steadily operate at 1.0 A over 100 h with negligible changes in the cell voltage (Figure 3h). More importantly, the volume of the NaOH electrolyte maintains almost unvaried after electrolysis, as demonstrated by the much smaller standard error (4.6 mL) than theoretical water consumption (40.3 mL) under the operation conditions (Figure S11, Supporting Information). Also, the corresponding pH value of the NaOH electrolyte exhibits negligible decrease after the long‐term stability test for 100 h under the electric field, indicative of the relatively constant NaOH concentration and the driving force (Δ*C*) for water transmembrane transport (Figure S12, Supporting Information). These results confirm the successful establishment of the water dynamic balance (*q*_influx_ = *q*_outflux_). Thus, the actual scenario of desired continuous DSS for H_2_ production at a given *i* value can be realized by readily adjusting the Δ*C* according to the water balance.

### 2.4 Degradation Mechanism of CEMs Operating in Natural Seawater

Having proved the operational feasibility of the continuous DSS system from stimulated seawater sources, possible interferences originating from Mg^2+^/Ca^2+^ ions in natural seawater should be also considered before allowing this system toward practical applications. The natural seawater from Qingdao Bay, China was taken as a practical example of the water source, which was compositionally analyzed to contain 12,715 mg L^−1^ of Cl^−^ ions (corresponding to 0.36 m of Cl^−^ ions), 125 mg L^−1^ of Mg^2+^ ions, and 10 mg L^−1^ of Ca^2+^ ions (Figure S13 and Table S2, Supporting Information). When using the Gore CEM as the separator between natural seawater and pure water, the concentrations of Mg^2+^/Ca^2+^ cations in pure water are lower than those in natural seawater by an order of magnitude after standing for 96 h (Figure S14, Supporting Information), suggesting the satisfactory interception of Gore CEM for divalent cations. To verify this, we then assembled DSS systems with Gore CEMs and other commercially available monovalent‐selective CEMs, including ASTOM Neosepta CIMS, FUMA Fumasep FKB‐PK‐130, and AGC Selemion CSO (denoted as Neosepta, Fumasep, and Selemion, respectively), as comparisons (see Table S1, Supporting Information, for details).

**Figure 4:**
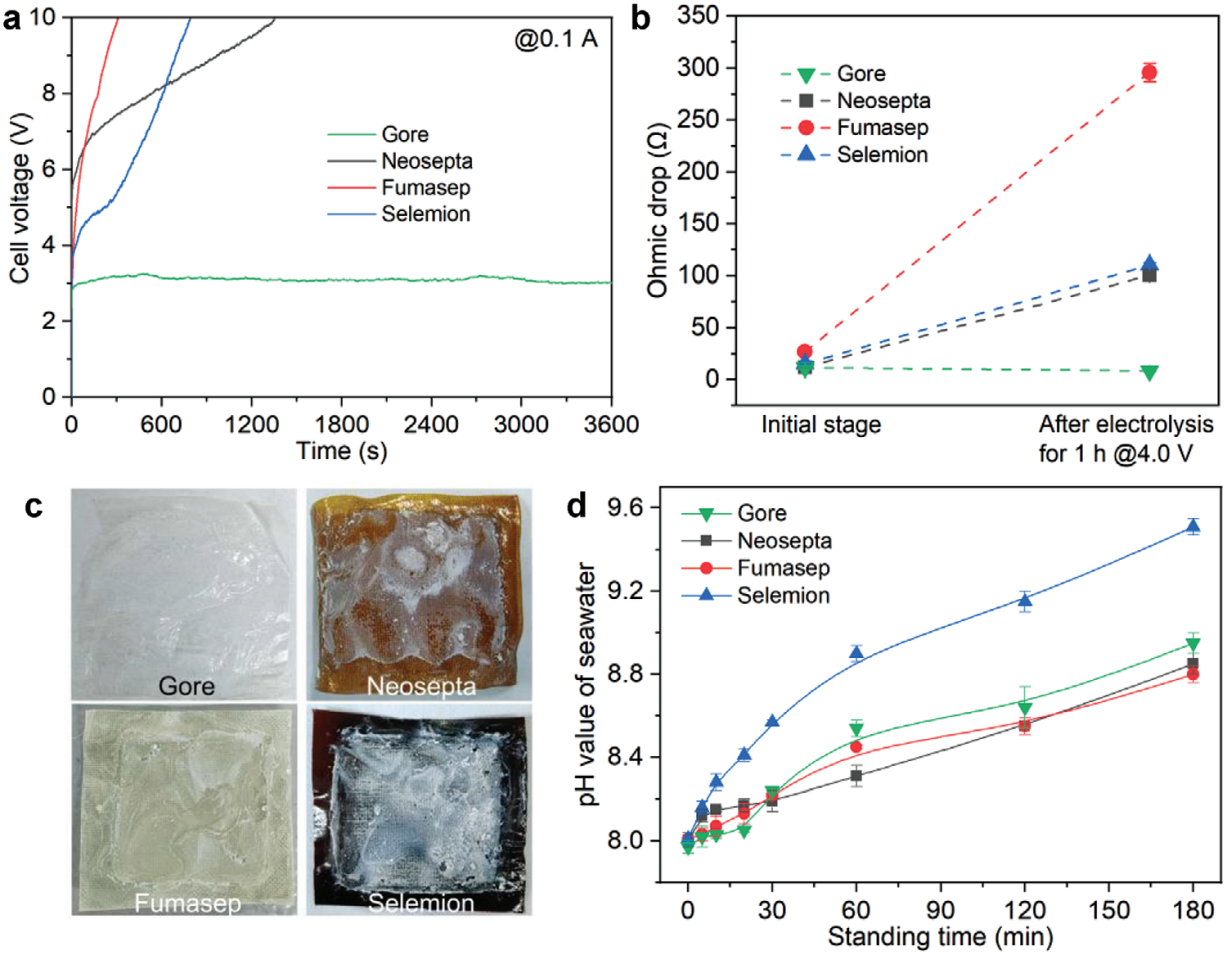
a) Galvanostatic electrolysis curves of the DSS systems assembled by different CEMs at 0.1 A (seawater chamber: natural seawater; electrolyte chambers: 1.0 m NaOH). b) Ohmic drops of the DSS systems at the initial stage and after potentiostatic (4.0 V) electrolysis for 1 h. c) Images of CEMs after potentiostatic electrolysis at 4.0 V for 1 h. d) pH value evolution of seawater (with a fixed volume) as a function of the standing time in the systems assembled by various CEMs, wherein another side of the testing cell is 1.0 m NaOH (pH = 13.8).

The galvanostatic electrolysis results in **Figure** **4**a show that the DSS system using Gore CEMs can stably run for 1 h with negligible changes in the cell voltage (maintaining at ≈3.0 V). When assembled with other CEMs (Neosepta, Fumasep, and Selemion), the corresponding DSS systems collapse at only around 1200 s, evidenced by the dramatically increased cell voltages (up to 10.0 V). This performance contrast becomes more pronounced at a higher current of 0.25 A, as three reference CEMs merely support the DSS system for 200 s while the Gore CEM can still endow the system with robust stability (Figure S15, Supporting Information). To decipher the underlying reason for this phenomenon, we then prolonged the operation time of DSS by adopting a relatively low cell voltage (4.0 V), which allowed us to track the changes of electrolyzer in inner structures and CEMs in physical properties. The results suggest that the DSS system with Gore CEMs can deliver a current of ≈0.2 A, which is more than double those of the reference systems at the same cell voltage (Figure S16, Supporting Information). After 1 h electrolysis, the DSS systems equipped with Neosepta, Fumasep, and Selemion CEMs encounter dramatic increases in the ohmic drop by 8.5 (from 11.7 to 100.3 Ω), 11.3 (from 26.7 to 295.8 Ω), 7.3 times (from 15.1 to 110.1 Ω), respectively, while no alteration of ohmic drop can be observed in the Gore CEM‐assembled case (Figure 4b). More importantly, there is no precipitation formed over the surfaces of Gore CEMs (Figure 4c), in contrast with the distinct white precipitations (mainly Mg(OH)_2_, Figure S17, Supporting Information) deposited on other three CEMs. Despite the obvious precipitation formation, the dissolved fractions of the other three CEMs were too low to be neglected after the long‐term electrolysis (Figure S18, Supporting Information), confirming that these membranes cannot be dissolved during the electrolysis process. Interestingly, the Mg(OH)_2_ precipitations dominantly locate on the internal surfaces of CEMs that directly contact with seawater (Figure S19, Supporting Information), indicating that the precipitation formation could be attributed to the minute amounts of OH^−^ leaking from the NaOH electrolyte to seawater through CEMs, rather than the migration of Mg^2+^ from seawater to electrolyte.

To unlock the reasons for this phenomenon, we further studied the OH^−^ permeation behavior of CEMs between natural seawater (30.0 mL) and 1 M NaOH solution (30.0 mL). Gradually elevated pH values of seawater are detected with the increase of the standing time regardless of the kind of CEM employed (Figure 4d), indicating that the adopted CEMs cannot resist the OH^−^ transport completely. To our surprise, for the most easily inactivated CEM (Fumasep, ≈300 s lifespan at 0.1 A, see Figure 4a), the corresponding increment in pH value of seawater is not the most conspicuous, which means that the OH^−^ permeation across CEMs is also not the main reason for the breakdown of DSS. Instead, the differences of the CEMs performed in DSS system are most likely associated with the surface properties of CEMs, for example, the existence of electron‐deficient sites that have been identified to be helpful for weakening the interplays between OH^−^ and Mg^2+^/Ca^2+^ ions, which remains to be further explored.^[^ ^18^, ^19^, ^43^, ^44^ ^]^

### 2.5 Practical Continuous DSS Operation for Natural Seawater

**Figure 5:**
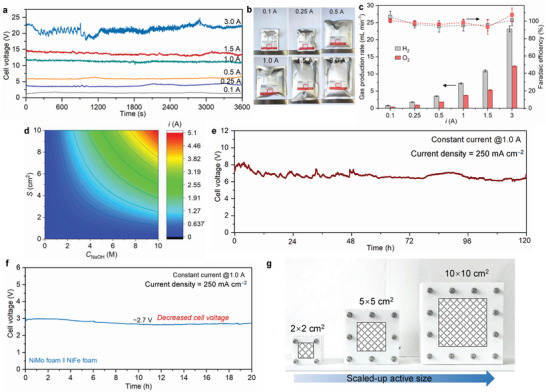
a) Galvanostatic electrolysis curves of continuous DSS as a function of current (NaOH concentration: 1.0 m). CEM: Gore. b) Images for the gas bags of H_2_ collected at different currents for 1 h. c) HER/OER FEs and the gas production rates of the DSS systems performed by galvanostatic electrolysis. d) Heatmap of the predicted *i* as a function of *C*_NaOH_ (*x* axis) and *S* (*y* axis) in a continuous DSS system. e) Galvanostatic electrolysis curve of continuous DSS performed at 1.0 A for 120 h, wherein the NaOH concentration is 2.4 m. f) Galvanostatic electrolysis curve of NiMo foam||NiFe foam system at 1.0 A for 20 h. g) Images for the scaled‐up electrolyzers with various membrane sizes of 2 × 2, 5 × 5, and 10 × 10 cm^2^, respectively, and their LSV curves.

Combining the above results, it is logically feasible to realize continuous DSS for natural seawater based on the water dynamic balance established in our electrolyzer. The galvanostatic electrolysis results reveal that the cell voltage is relatively stable in the current range of 0.1 to 1.5 A, followed by a certain degree of fluctuation at 3.0 A due to the furious gas turbulence (**Figure** **5**a). A visible increase of the H_2_ volume is confirmed along with the increase in current (Figure 5b). Calculated H_2_ and O_2_ production rates also gradually augment from 0.1 to 3.0 A, (from 0.8 to 21.3 mL min^−1^ for H_2_; from 0.34 to 12.3 mL min^−1^ for O_2_), and advantageously, the corresponding FEs remain nearly 100% (Figure 5c).

To pursuit the practical application of this electrolyzer engineering, we then sought to build a continuous DSS system with natural seawater as the water source. Similarly, we first studied the relationship between *q*_influx_ and NaOH electrolyte concentration (denote as *C*_NaOH_) following Fick's law. The fitting curve of *q*_influx_ and *C*_NaOH_ also presents a good linear relationship with the equation of *q*_influx_ = 4.8 × 10^−6^ *SC*_NaOH_ (*R* ^2^ = 0.99) (Figure S20, Supporting Information). Thus, the conditions for a continuous DSS system (*q*_influx_ = *q*_outflux_) can be described as follows.

(8) $$i=\;0.051SC_{\mathrm{NaOH}}$$

This relationship involving *i*, *S*, and *C*_NaOH_ for a continuous DSS system is further visualized and presented in Figure 5d, indicating that *i* is also positively related to *S* and *C*_NaOH_ in a continuous DSS system, which can be used to determine the *C*_NaOH_ at a given *i* for H_2_ production. For instance, for the adopted electrolyzer with a membrane size of 2 × 2 cm^2^ (corresponding to *S* = 8 cm^2^), the theoretical relationship between *i* and *C*_NaOH_ can be specifically formulated as *i* = 0.41*C*_NaOH_. Thus, to enable continuous DSS with a practically attractive current of 1.0 A, a 2.4 m NaOH electrolyte was adopted. The established DSS system can run stably at 1.0 A over 100 h with a slight change of the voltage (maintaining at ≈7.0 V, see Figure 5e). After the galvanostatic electrolysis for 120 h, the ohmic drop of the system remains almost unchanged, albeit with the presence of a small amount of precipitation formed over the CEM surface (Figure S21, Supporting Information). Furthermore, no obvious changes can be observed for the attenuated total reflection (ATR) spectra of the Gore membrane before and after the electrolysis (Figure S22, Supporting Information); meanwhile, the dry weight of the membrane remains almost unchanged before and after the electrolysis with a negligible dissolved fraction of 1.28% (Figure S23, Supporting Information), fully confirming that the Gore membrane cannot be dissolved during the electrolysis process. In addition, Ni foams also maintain their initial crystalline structure (Figure S24, Supporting Information). More meaningfully, the volume of the NaOH electrolyte is confirmed to be 157.5 (± 4.5) mL after electrolysis (Figure S25, Supporting Information), which approximately equals to the initial value (160.0 mL), evidencing the reliability of the proposed water dynamic balance (*q*_influx_ = *q*_outflux_).

To verify the practicability of this electrolyzer, we further optimized the electrolyzer with more active catalysts such as commercial NiMo and NiFe foams as the cathode and anode catalysts, respectively. It is revealed that the NiMo foam||NiFe foam system can also render ≈100% FEs for HER and OER (Figure S26, Supporting Information), further demonstrating the practical upgradability of our electrolyzer. More impressively, the cell voltage of the NiMo foam||NiFe foam system is significantly lower than that of the original Ni foam||Ni foam system (Figure 5f); accordingly, the energy consumption of the NiMo foam||NiFe foam system is calculated to be ≈6.2 kWh Nm^−3^, which is comparable to the state‐of‐the‐art reports (Table S3, Supporting Information). Especially in terms of the intrinsic catalysis activity and the ion/water transport process, there is still much room to further the reduce energy consumption of this electrolyzer platform by coupling advanced catalyst or membrane materials.

Also, to validate the scalability of our DSS system design, we extended the membrane size of electrolyzer (such as 5 × 5 cm^2^ and 10 × 10 cm^2^) (Figure 5g). The LSV analysis reveals that the currents (at the same voltage) of the scalable DSS systems display an increased tendency with the increase of membrane size, which is mainly attributed to its gradually decreased ohmic drop (Figures S27 and S28, Supporting Information). This phenomenon is also in consistent with the voltage response of the galvanostatic electrolysis at 1.0 A (Figure S29, Supporting Information). The energy efficiency of the electrolyzer with a membrane size of 10 × 10 cm^2^ is calculated as high as 33.2%; meanwhile, the scalable DSS systems also deliver impressive selectivity for HER and OER at a high current of 1.0 A, with FEs approaching to nearly 100% (Figure S30, Supporting Information). More performance benefits from upscaling can be anticipated with the integration of better materials (catalysts, membranes, etc.) and should derisk the use of this electrolyzer concept for commercial‐scale H_2_ production in industrial settings. Of note, although the DSS technology is still in its infancy stage, it would become an appealing pathway for green H_2_ production in the future by the further catalyst design, system optimization, etc., especially for some special application scenarios (such as ships, coastlines, etc.).

## 3 Conclusions

In summary, we have reported an electrolyzer engineering strategy for ampere‐level green H_2_ production from natural seawater without assistance by specially designed catalysts. The core technology is the dual‐CEM architecture integrated with a circulatory electrolyte design, which avoids the precipitate formation and chlorine corrosion while maintaining a constant concentration gradient driving force for transmembrane (seawater to electrolyte) water capture. The specific relation of water transport balance in continuous DSS system (e.g., a membrane size of 2 × 2 cm^2^) involving *i* and *C*_NaOH_ was successfully established (*i* = 0.41*C*_NaOH_). Such a continuous DSS system equipped with readily available Ni foams can run at an industry‐relevant current of 1.0 A over 100 h with a H_2_ production rate of 7.5 mL min^−1^ (*C*_NaOH_ = 2.4 m). Factoring its durability and scalable feasibility, this electrolyzer concept could in principle be extended beyond seawater to other low‐grade water feeds (e.g., brine, wastewater, etc.). It is worth noting that, to further reduce the ohmic resistance of this system, a zero‐gap electrolyzer with minimized distance between the anode and cathode deserves to be further developed in the future. Considering the challenges likely to emerge at industry level, further upgradation guided by industrially realized technologies such as chlor‐alkali process (with mature crafts, membranes, etc.), together with directional development of CEMs for improved permselectivity/stability in seawater environments (potentially through surface chemical modifications), should also be accompanied to move this DSS technology closer to the market.

## 4 Experimental Section

### Chemicals and Materials

NaOH (Sigma‐Aldrich, >98%), NaCl (Macklin, 99.5%), D_2_O (Macklin, 99.9 atom% of D), H_2_^18^O (Aladdin, 97 at% of ^18^O), NaClO aqueous solution (Macklin, 0.1 m), and 4‐amino‐N,N‐diethylaniline monohydrochloride (Macklin, 98%) were used without purification. The CEMs include GORE‐SELECT Gore M788.12 (W. L. Gore & Associates, America) and FUMA Fumasep FKB‐PK‐130 (FuMa Tech., Co., Ltd., Germany) were provided by SCI Materials Hub. The ASTOM Neosepta CIMS (ASTOM Co., Ltd., Japan) and AGC Selemion CSO (AGC Co., Ltd., Japan) were purchased from Huamo Technology Co., Ltd. The Ni foam (thickness: 300 µm) was provided by Kunshan Guangjiayuan Electronic Materials.

### Characterizations

The morphology and structure of Ni foam were investigated by scanning electron microscopy (SEM, Hitachi S‐4800) and X‐ray diffraction (XRD, Bruker D8 ADVANCE) using Cu Kα radiation (*λ* = 1.5406 Å). The concentrations of Ca^2+^, Mg^2+^, and Cl^−^ ions in seawater and the samples were determined by ion chromatography (Thermo Fisher Scientific, ICS‐5000). The absorbances of the samples at various wavelengths were collected by an ultraviolet–visible (UV–Vis) spectrophotometer (Hitachi, U‐4100).

### Transmembrane Water Migration

The migration behavior of water across the membrane was investigated by determining the volume of water within a certain time under ambient conditions.^[^ ^35^ ^]^ Here a homemade H‐type cell with a volume of 5.0 mL was adopted, in which the two chambers of the cell were separated by the CEM. Specifically, add 5.0 mL of simulated seawater/natural seawater and 5.0 mL of NaOH solutions with known concentrations (1.0, 3.0, 6.0 m) into the two chambers, respectively. After standing for 15 h, the above solutions were taken out from the chambers, and the volume of the NaOH solution was determined by a measuring cylinder. According to the change in volume of the NaOH solution before and after the standing process (Δ*V*), the migration rate of water towards the CEM was obtained. Meanwhile, the relationships between *q*_influx_ and Δ*C*/*C*_NaOH_ were also determined by the linear fitting.

### Assembly of DSS System

The DSS electrolyzer has a symmetrical structure with a seawater chamber in the middle and electrolyte (concentrated NaOH solution) chambers on two sides, where the chambers were spatially separated by two CEMs. Commercial Ni foams were used both as cathode and anode. The sizes of CEMs and Ni foams were 2 × 2 cm^2^, which were fixed at the frames of chambers with waterproof tapes. The simulated seawater or seawater were continuously purged into the seawater chamber by a pump with a rate of 10 mL min^−1^. The NaOH electrolyte was cycled by pumps with a rate of 10 mL min^−1^. Ti foams were selected as the current collectors for the anode and cathode. The scaled‐up DSS systems were also assembled according to similar procedures with different sizes of CEMs and Ni foams (5 × 5 or 10 × 10 cm^2^).

### Electrochemical Tests

The electrochemical tests were performed in a two‐electrode configuration with an electrochemical workstation (CHI440A, CH Instruments) and DC power supply (A‐BF, SS‐L650SPD, 60 V/5 A; resolutions: 1 mV/0.1 mA). Linear sweep voltammetry (LSV) was recorded at a scan rate of 100 mV s^−1^ without *iR*‐compensation. The galvanostatic/potentiostatic tests were conducted without *iR*‐compensation. The ohmic drop of the electrolysis system was determined with the electrochemical workstation three times and averaged.

### DEMS Characterization

The DEMS measurements were conducted with an online mass spectrometry (Hiden HPR20), which was electrochemically controlled by a Land test system. The electrochemical device with CEM/Ni foam sizes of 2 × 2 cm^2^ was assembled and employed in the tests. 1.5 mL of aqueous NaOH solution (1.0 m) was added into the cathode and anode chambers, respectively. 1.5 mL of simulated seawater (0.6 m of NaCl D_2_O or H_2_^18^O solutions) was added into the middle seawater chamber. The Ar gas (99.999%) with a rate of 1.0 mL min^−1^ was adopted as the carrier gas for the mass spectrometry system. A cold trap was deployed between the electrochemical device and mass spectrometry to prevent the moisture of the electrolyte from entering the mass spectrometry. The gas tightness was first examined before testing, and the corresponding DEMS measurement was performed until only the signal of Ar can be detected. Then the DEMS measurements for the gas products from cathode/anode chambers were triggered by a constant current of 10 mA for 20.0 min when the baselines of the signals were smooth and steady.

### In Situ Raman Measurements

In situ Raman experiments were conducted on a confocal Raman microscope (HORIBA LabRAM Odyssey). A H‐type cell with a three‐electrode system was employed for electrochemical measurements, where a Hg/HgO (filled with 1 m KOH) and Pt wire were used as the reference and counter electrodes, respectively, whose chambers were separated by an anion exchange membrane. Ni foams (geometric area: 1.0 cm^2^) were used as the working electrodes for HER and OER processes. 1 M NaOH aqueous solution was selected as the electrolyte, which was swept for 30 min by Ar gas before tests. The Raman spectra were collected at various applied potentials in the range of 200–1000 cm^−1^ (acquisition time: 20 s, accumulations: 4, hole: 1000, ND filter: 10%).

### Determination of HClO/ClO^−^

The concentration of HClO/ClO^−^ was determined by a modified DPD method based on the fact that the DPD can react with HClO/ClO^−^ to form a pinkish color complex, which can be measured by spectrophotometry.^[^ ^23^ ^]^ In a typical run, 3 mL of the after‐reaction electrolyte (or diluted electrolyte with deionized water) was taken from the cell, followed by adding 20 µL of 1 m H_2_SO_4_ solution and 1 mL of 4‐amino‐N,N‐diethylaniline monohydrochloride aqueous solution (0.05 g mL^−1^). Next, the absorbance of the above solution was quickly measured by a UV–Vis spectrophotometer in a wavelength range of 400–650 nm. The standard curve was constructed by fitting the HClO/ClO^−^ concentrations of reference solutions (0, 20, 40, 60, 80, and 100 mL) versus corresponding absorbance values at 550 nm. The obtained fitting curve (*y* = 0.0143*x* + 0.04, *R* ^2^ = 0.99) exhibited a good linear relation and was employed to measure the unknown concentrations of HClO/ClO^−^ in the post‐reaction electrolytes.

### Determination for the Dissolved fraction of the Membranes

The dissolved fraction of the membrane was calculated as follows.^[^ ^45^ ^]^

(9) $$\mathrm{Dissolved}\;\mathrm{fraction}\;=\;\frac{m_{1}-m_{2}}{m_{1}}\;\times \;100\%$$

where *m*_1_ and *m*_2_ are the dry weights before and after the electrolysis test, respectively. Note that the membranes used in the long‐term potentiostatic electrolysis were washed using acid solution (0.1 m HCl) and deionized water, respectively, followed by vacuum drying at 80 °C for 12 h.

### Faradaic Efficiency

The generated gases (H_2_ and O_2_ gases) during the electrolysis were collected using gas bags, and the corresponding volumes were determined by syringes with different capacities and minimum division values (MDVs) (10.0 mL, MDV: 0.2 mL; 20.0 mL, MDV: 0.5 mL; 50.0 mL, MDV: 1.0 mL; 100.0 mL, MDV: 2.0 mL). According to the volume of the gas product and the applied current, the Faradaic efficiencies (FEs) of H_2_ and O_2_ can be calculated as follows:

(10) $$FE_{H_{2}}\;=\;\frac{V/V_{m}}{it/2F}\times 100\%$$

(11) $$FE_{O_{2}}\;=\;\frac{V/V_{m}}{it/4F}\times 100\%$$

where *V* is the volume (L) of the gas product, *V*_m_ is the standard molar volume at room temperature (24.5 L mol^−1^), *i* is the current (A), *t* is the electrolysis time (s), and *F* is the Faraday constant (96485.3 C mol^−1^).

### Energy Efficiency and Energy Consumption

The energy efficiency (EE) of the DSS system for H_2_ production was calculated by the following equation:^[^ ^46^ ^]^

(12) $$\mathrm{EE}\;\left(\%\right)=\frac{1.23}{\mathrm{Cell}\;\mathrm{voltage}}\times FE_{H_{2}}$$

To evaluate the electricity expenses of the proposed DSS system for H_2_ production, the corresponding average energy consumption (EC) was also calculated. The electric energy (*W*, Wh) consumed by the electrochemical system for H_2_ production was calculated as follows.^[^ ^26^ ^]^

(13) W=∫UIdt

Then the EC can be further calculated according to the following equation.

(14) $$EC\left(\mathrm{kWh}\;Nm^{-3}\right)\;=W/V$$

where *V* (L) is the experimentally determined volume of generated H_2_ gas.

## Conflict of Interest

The authors declare no conflict of interest.

## Supporting information

Supporting Information

## Data Availability

The data that support the findings of this study are available from the corresponding author upon reasonable request.
