# Supplementary material for: A Dual‐Cation Exchange Membrane Electrolyzer for Continuous H2 Production from Seawater
Source: Adv Sci (Weinh). 2024 Apr 3;11(25):2401702. doi: 10.1002/advs.202401702 (PMC11220719; doi:10.1002/advs.202401702)
Supplement: Supplementary file 1 — Supporting Information [file ADVS-11-2401702-s001.pdf]

ADVANCED SCIENCE
Open Access

Supporting Information

for *Adv. Sci.*, DOI 10.1002/advs.202401702

A Dual-Cation Exchange Membrane Electrolyzer for Continuous H_2 Production from Seawater

Yongwen Ren, Faying Fan, Yaojian Zhang, Lin Chen, Zhe Wang, Jiedong Li, Jingwen Zhao, Bo Tang* and Guanglei Cui**

Supporting Information

A dual-cation exchange membrane electrolyzer for continuous H_2 production from seawater

Yongwen Ren, Faying Fan, Yaojian Zhang, Lin Chen, Zhe Wang, Jiedong Li, Jingwen Zhao*, Bo Tang*, and Guanglei Cui*

Y. Ren, F. Fan, Y. Zhang, L. Chen, Z. Wang, J. Li, J. Zhao, G. Cui

Qingdao Industrial Energy Storage Research Institute, Qingdao Institute of Bioenergy and Bioprocess Technology, Chinese Academy of Sciences, Qingdao 266101, China.

Shandong Energy Institute, Qingdao 266101, China.

Qingdao New Energy Shandong Laboratory, Qingdao 266101, China.

E-mail: zhaojw@qibebt.ac.cn; cuigl@qibebt.ac.cn

B. Tang

Laoshan Laboratory, Qingdao 266237, China.

Email: btang@qnlm.ac (B. Tang)

G. Cui

School of Future Technology, University of Chinese Academy of Sciences, Beijing100049, China

Supplementary Figures

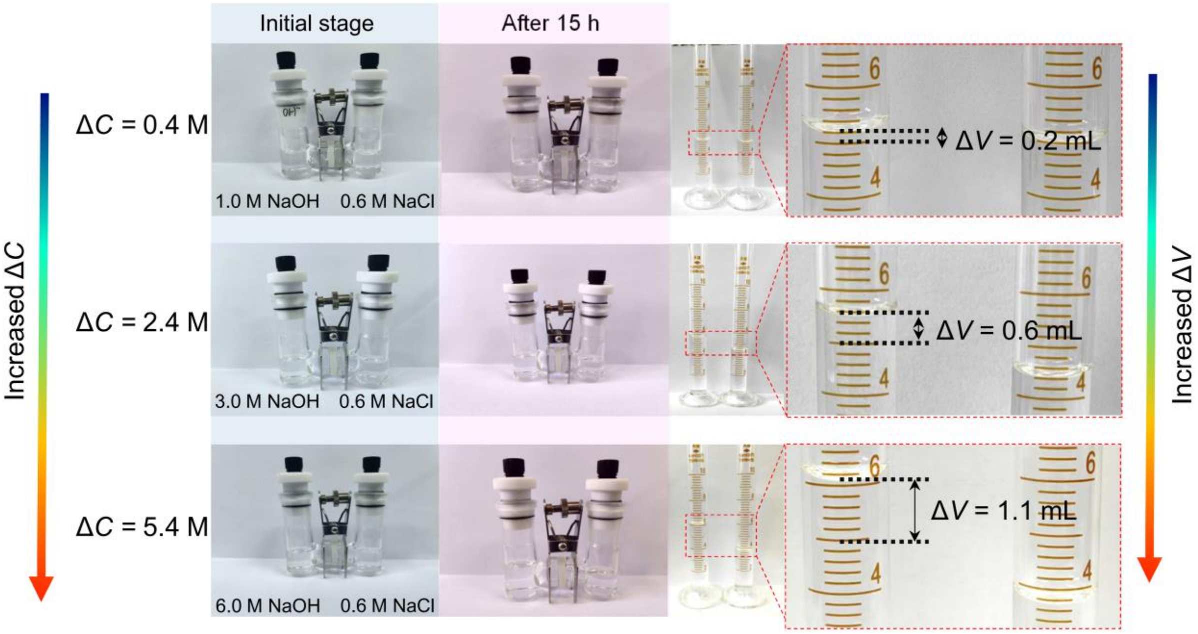

Figure S1. Determination of the relationship between q_{influx} and Δ*C*. Images of the H-type cell and the change of water in volume for water migration tests at the initial stage and after standing under ambient conditions for 15 h. To ensure the repeatability of the measurement, each test at a Δ*C* was conducted for three times.

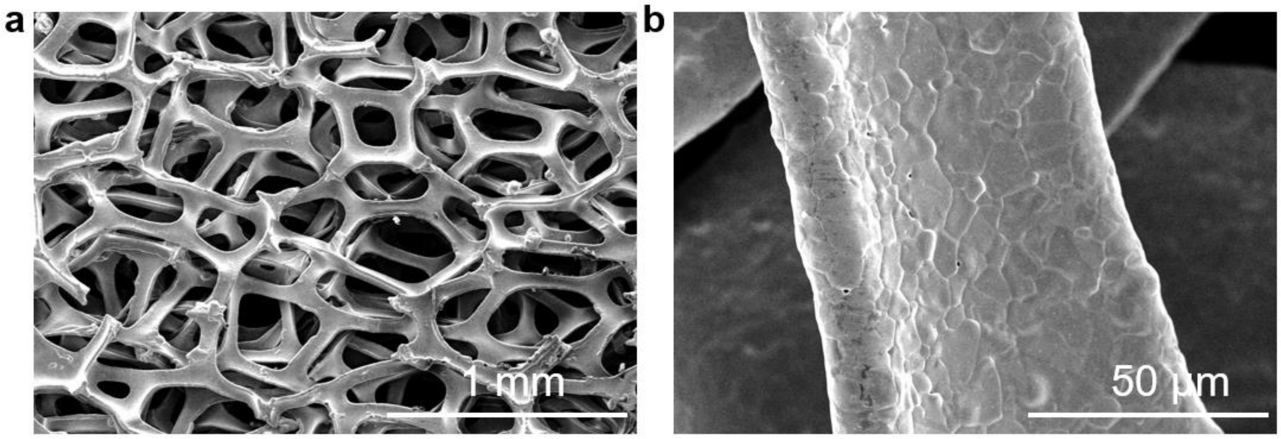

Figure S2. (a and b) SEM images for commercial Ni foam.

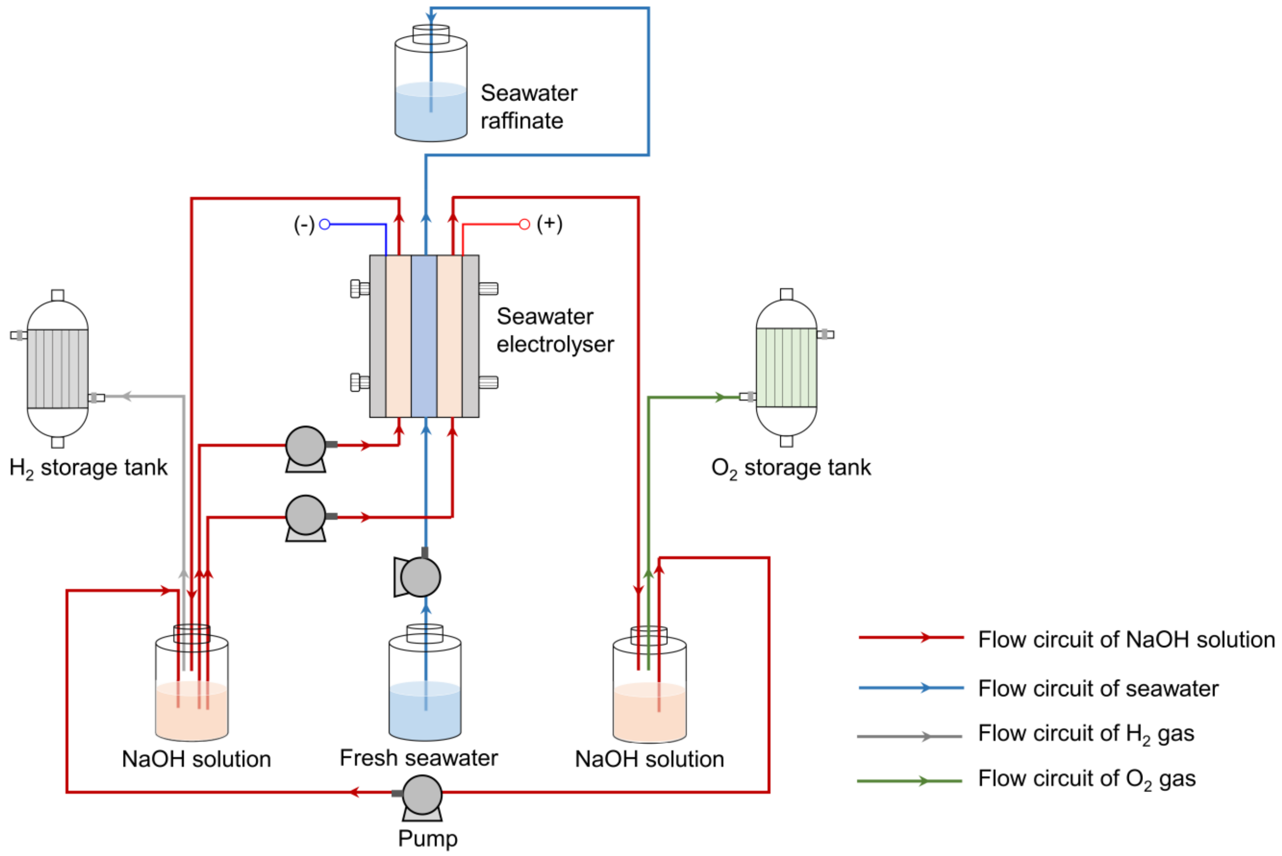

Figure S3. Schematic of the continuous DSS system for H_2 production.

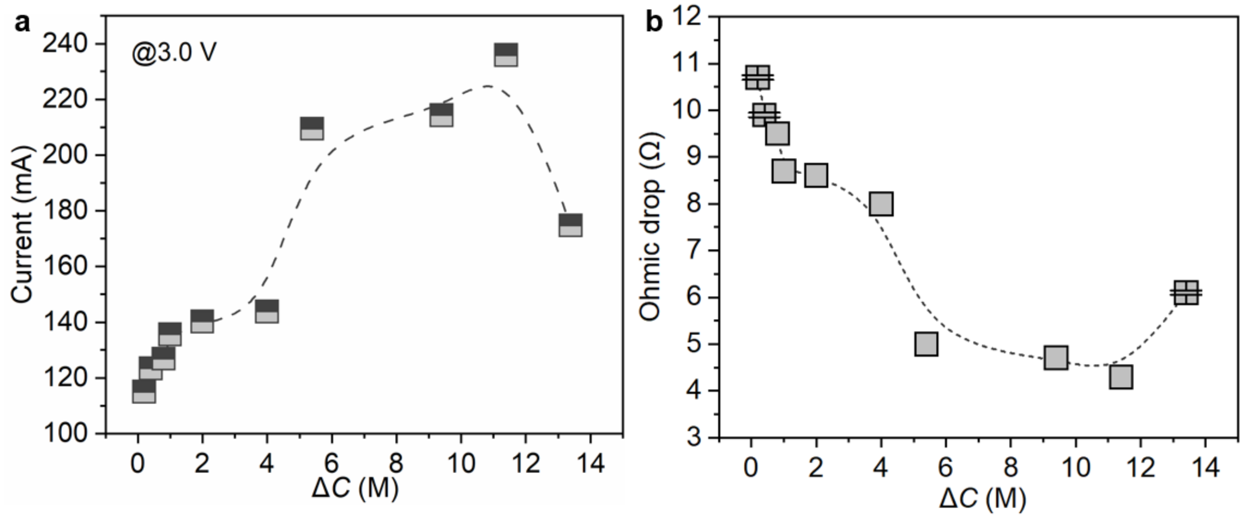

Figure S4. a, The current at a cell voltage of 3.0 V as a function of Δ*C*. The data is derived from the corresponding LSV curves. **b**, The Ohmic drop of the electrochemical system as a function of Δ*C*. Each test at a Δ*C* was conducted three times, and the results were averaged.

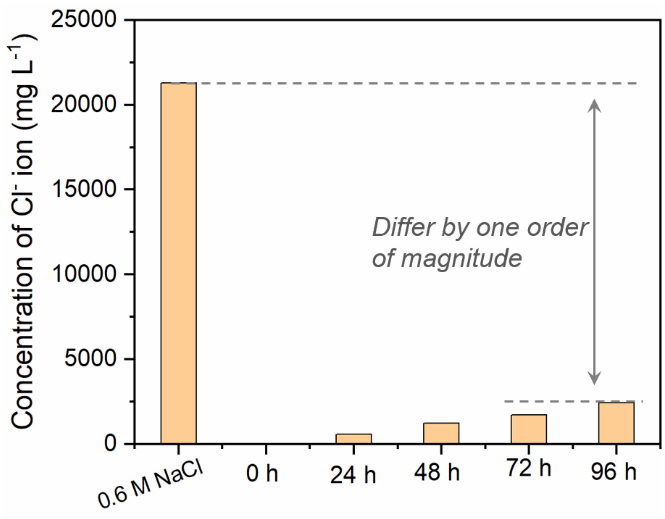

Figure S5. Variation for Cl^- ion concentration of pure water in the chamber of the H-type cell, wherein another chamber is filled with 0.6 M NaCl solution. The volume of the H-type cell is 30.0 mL, and the two chambers are separated by Gore CEM. The concentration of Cl^- ion is determined by ion chromatography. The results indicate that a certain amount of Cl^- ions can pass through the CEM after 96 h, but the corresponding concentration is still less than that of the 0.6 M NaCl by one order of magnitude.

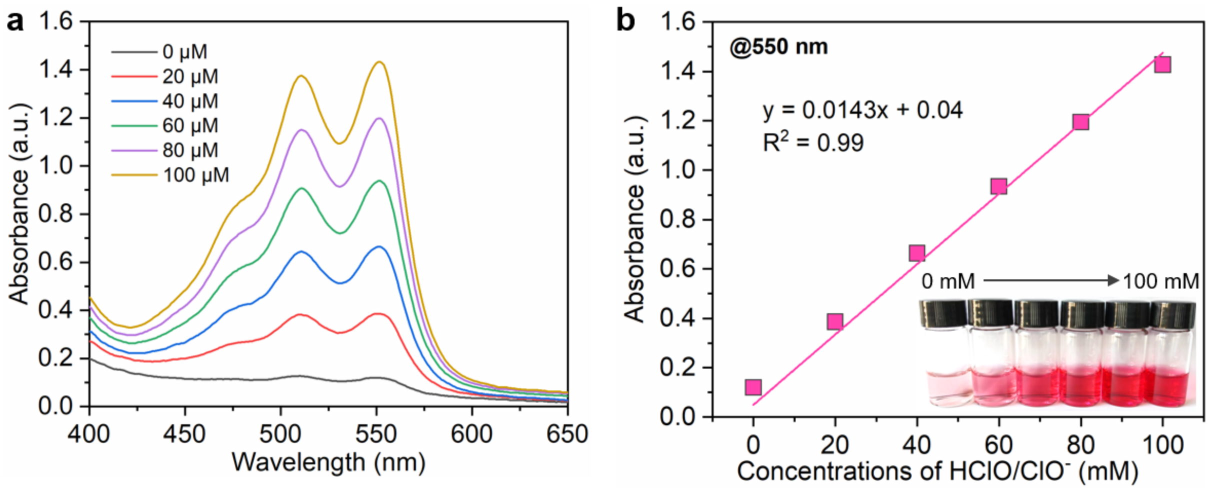

Figure S6. UV-Vis absorbance spectra (a) and corresponding fitting curve (b) of the modified DPD assay for determining $HClO/ClO^-$ with known concentrations of 0, 20, 40, 60, 80, and 100 mM, wherein the fitting curve was constructed with the absorbances of samples at 550 nm and the corresponding concentrations. The insert is the images for the samples stained with DPD.

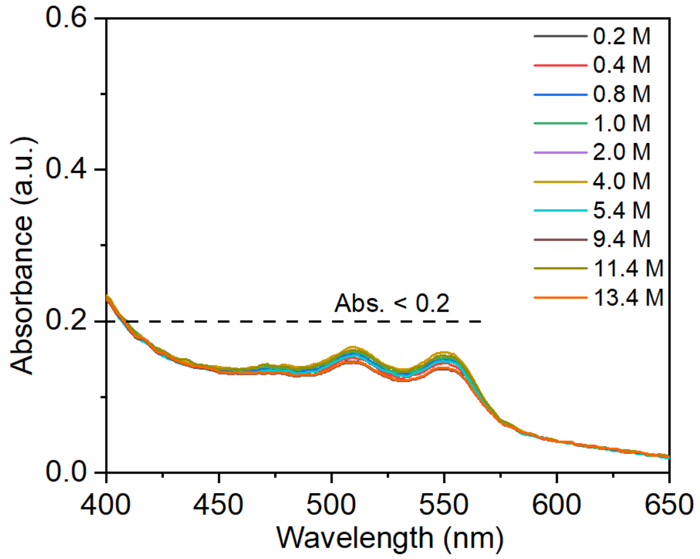

Figure S7. UV-Vis absorbance spectra for the electrolytes stained with DPD after the electrolysis at 0.25 A for 1 h in the range of Δ*C* from 0.2 to 13.4 M. The absorbances of electrolytes at 550 nm are less than 0.2, indicating that no HClO/ClO⁻ products exist in the electrolytes.

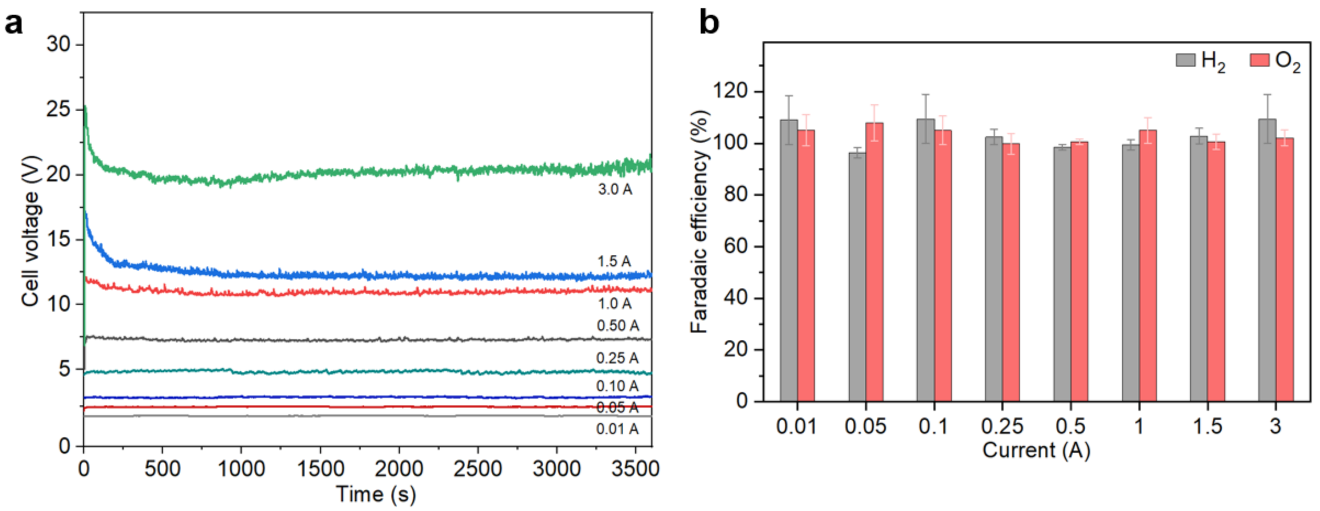

Figure S8. **a**, Galvanostatic electrolysis curves of the DSS system with simulated seawater at different currents with the increase of electrolysis time. Cathode/anode: Ni foams. NaOH concentration: 1.0 M. CEM: Gore. Δ*C* = 0.6 M. **b**, The corresponding FEs of HER and OER during the galvanostatic electrolysis at different currents for 1 h.

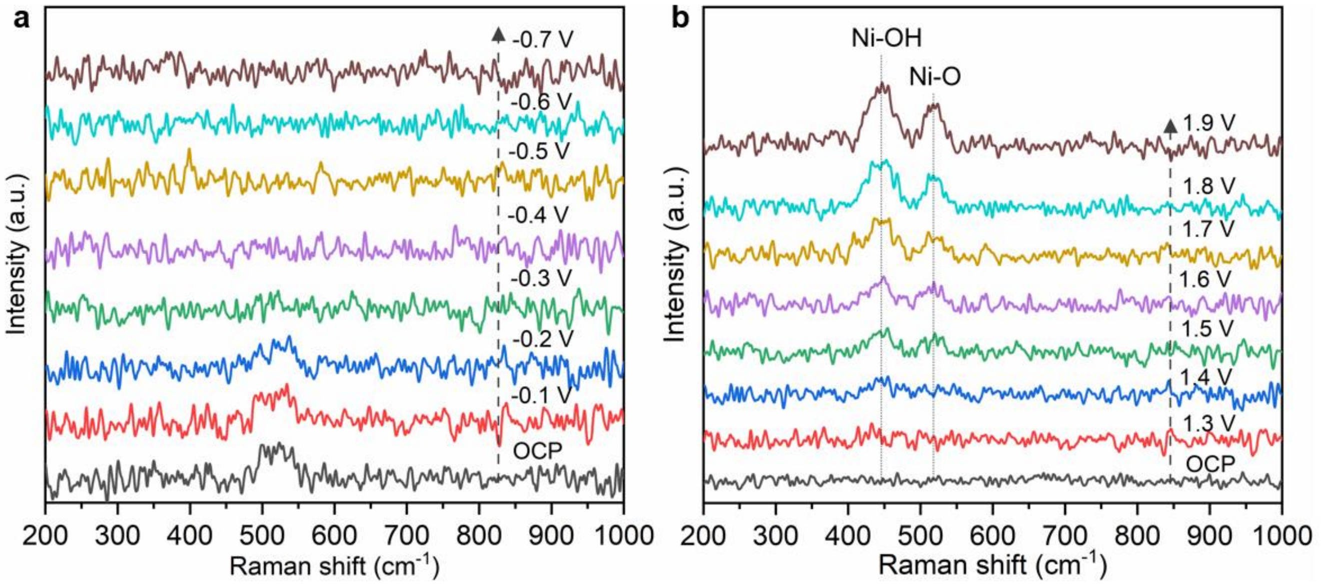

Figure S9. *In situ* Raman spectra of Ni foams for HER (a) and OER processes (b) performed in 1.0 M NaOH under various potentials (*vs* RHE), respectively.

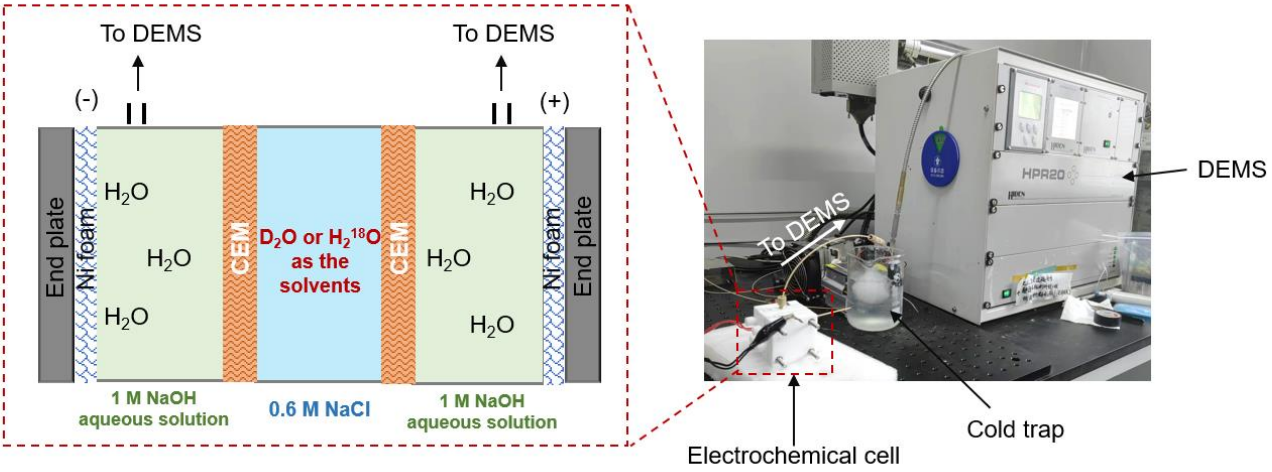

Figure S10. Image of the DEMS test system and the corresponding illustration for the adopted electrochemical device.

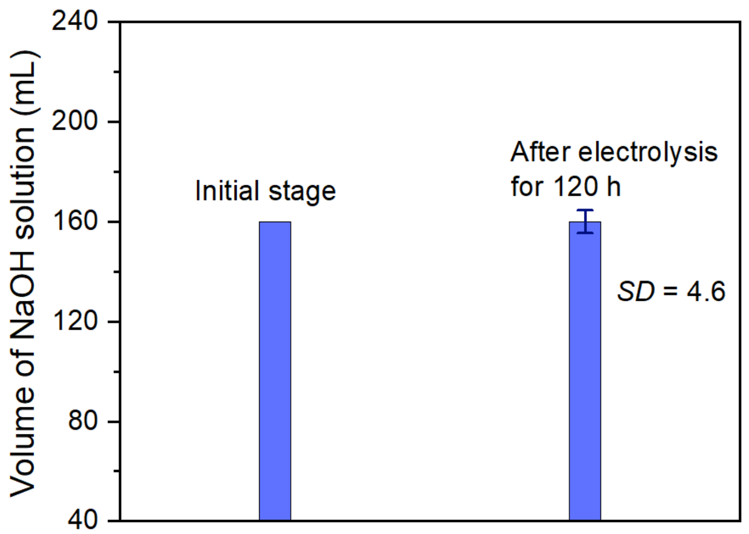

Figure S11. The volume of NaOH solution (2.9 M) before and after the constant current electrolysis at 1.0 A for 120 h. The test is conducted three times, and the corresponding standard error (*SD*) is calculated to be 4.6 mL.

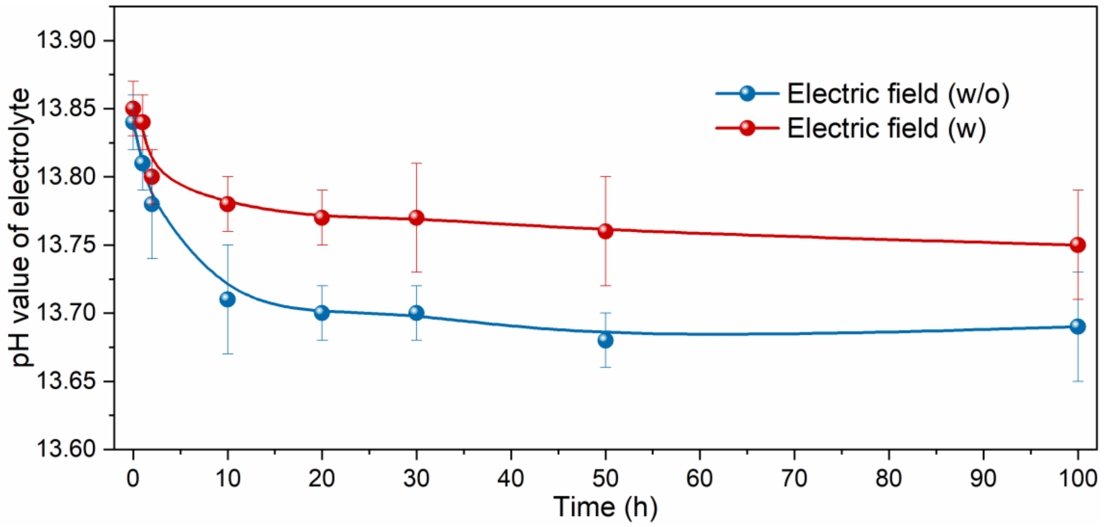

Figure S12. pH value of NaOH electrolyte (1.0 M) in the dual-CEM three-compartment electrolyzer as the function of time with/without applied electric field (galvanostatic electrolysis at 1.0 A), wherein the seawater was continuously purged into the seawater chamber. Without the applied electric field, the pH value of NaOH electrolyte slightly reduces with the increase of the time (initial stage: 13.84; after 100 h: 13.69), indicative of the slight decrease of NaOH concentration. However, in the practical system (with the applied electric field), the reduction range of NaOH pH value becomes smaller in contrast to the case without electric field (ΔpH = 0.10, 0.15, respectively), meaning that the electric field-enabled Na^+ migration can weaken the concentration gradient-enabled Na^+ diffusion to some degree. This is most likely due to the competitive Na^+ permeation in the finite ion channels. Thus, the NaOH concentration can maintain relatively constant during the electrolysis.

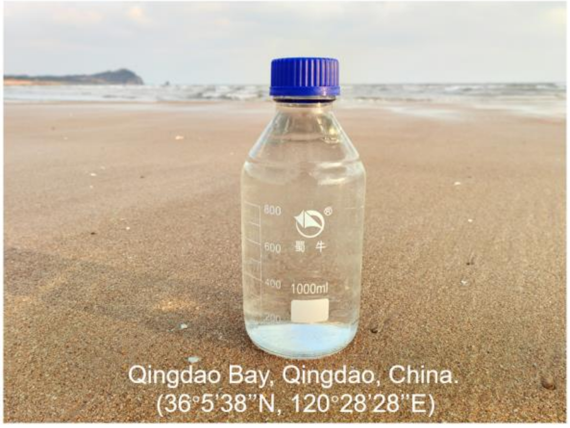

Figure S13. The optical image of the natural seawater from Qingdao Bay, Qingdao, China. Before use, the insoluble impurities of natural seawater were removed by filtering with polyethersulfone (PES) filtration membrane (pore size: 0.45 μm, ANPEL Laboratory Technologies).

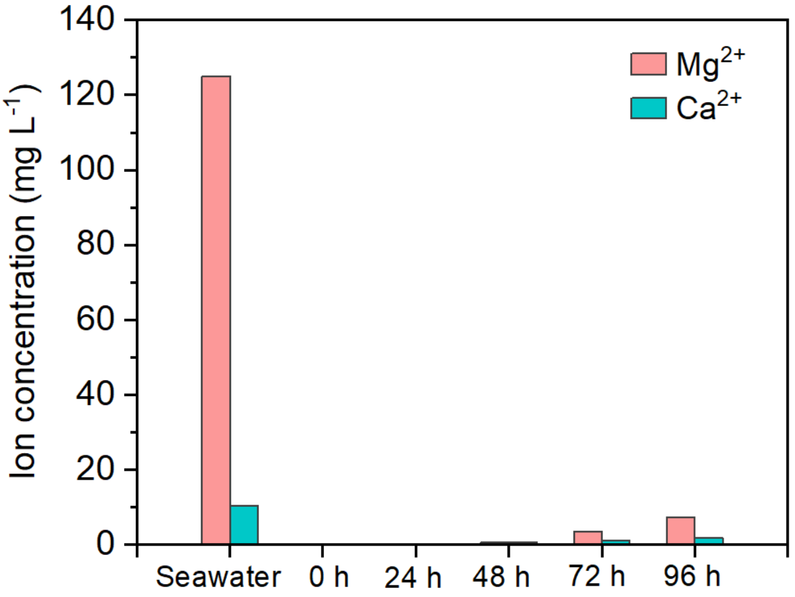

Figure S14. The change for Mg^{2+}/Ca^{2+} ion concentration of pure water in the chamber of the H-type cell, wherein another chamber is filled with natural seawater. The volume of the H-type cell is 30.0 mL, and the two chambers are separated by Gore CEM. The concentration of Mg^{2+}/Ca^{2+} ions were determined by ion chromatography. The results indicate that very low concentrations of Mg^{2+}/Ca^{2+} ions can be detected in the pure water after standing for 96 h.

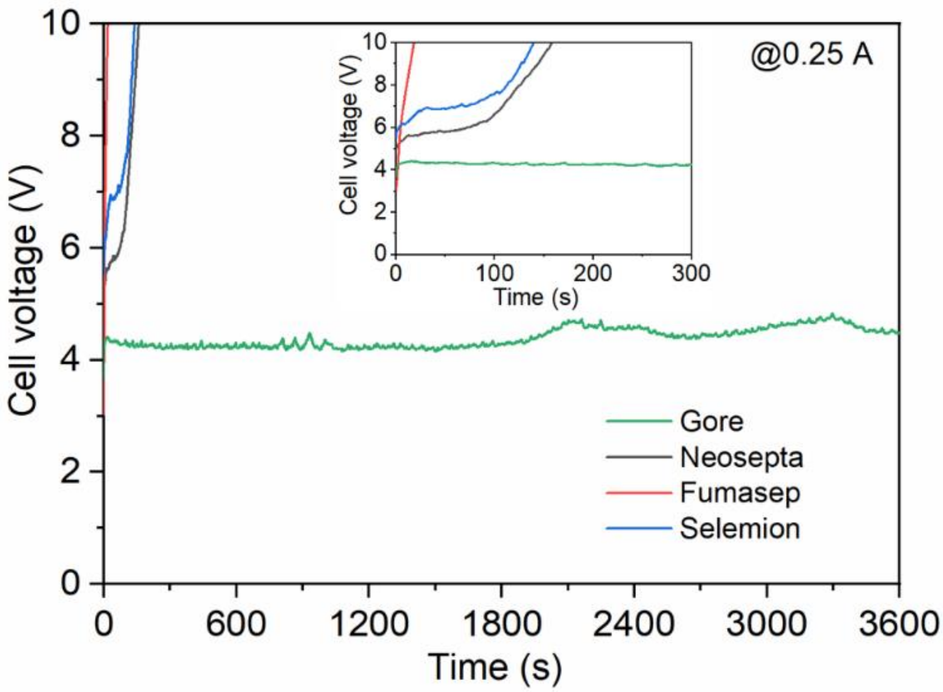

Figure S15. Galvanostatic electrolysis curves of the systems assembled with different CEMs at 0.25 A. NaOH concentration: 1.0 M.

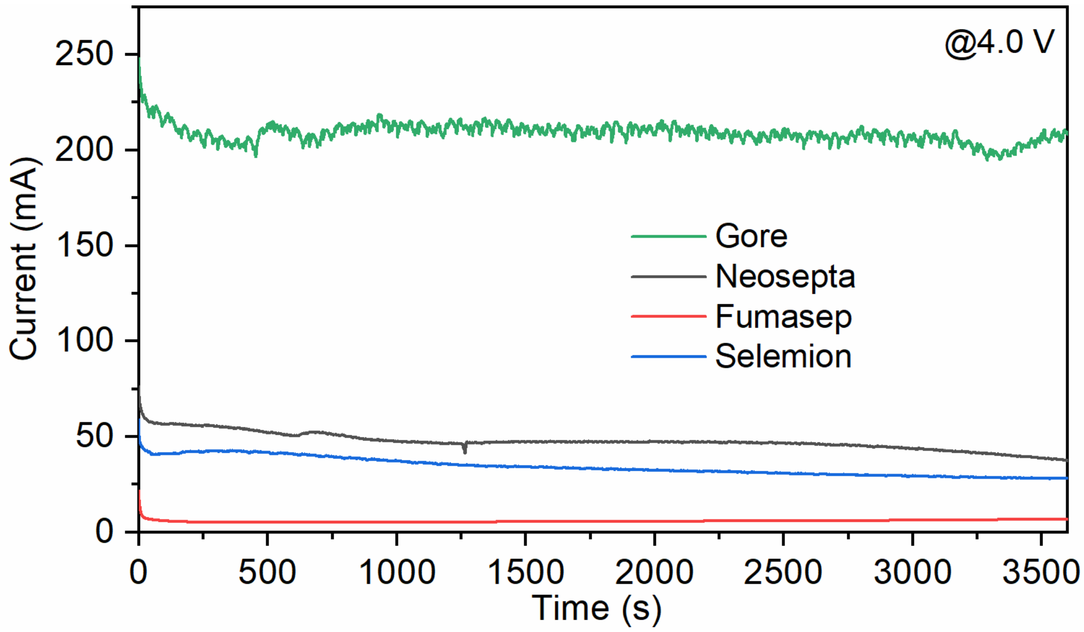

Figure S16. Galvanostatic electrolysis curves of the DSS systems assembled with different CEMs at 4.0 V for 1 h.

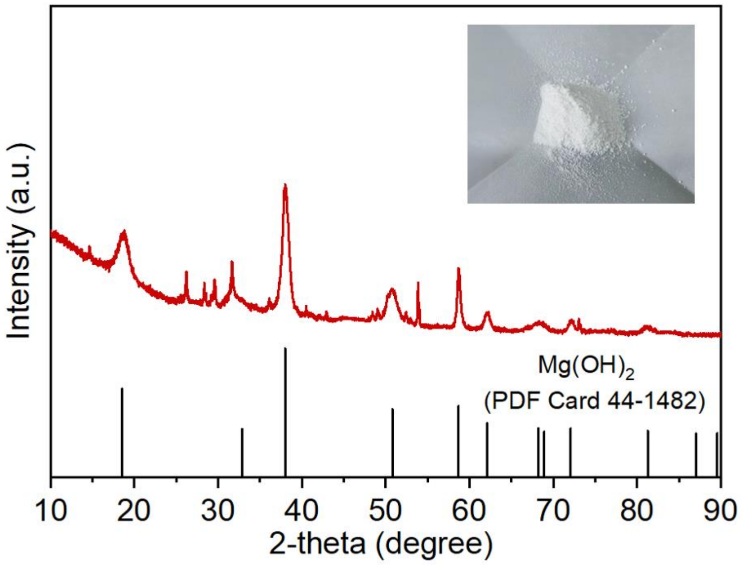

Figure S17. X-ray diffraction (XRD) pattern for the white precipitation over the Selemion CEM, wherein the precipitation was collected by constant voltage electrolysis at 4.0 V for 3 h.

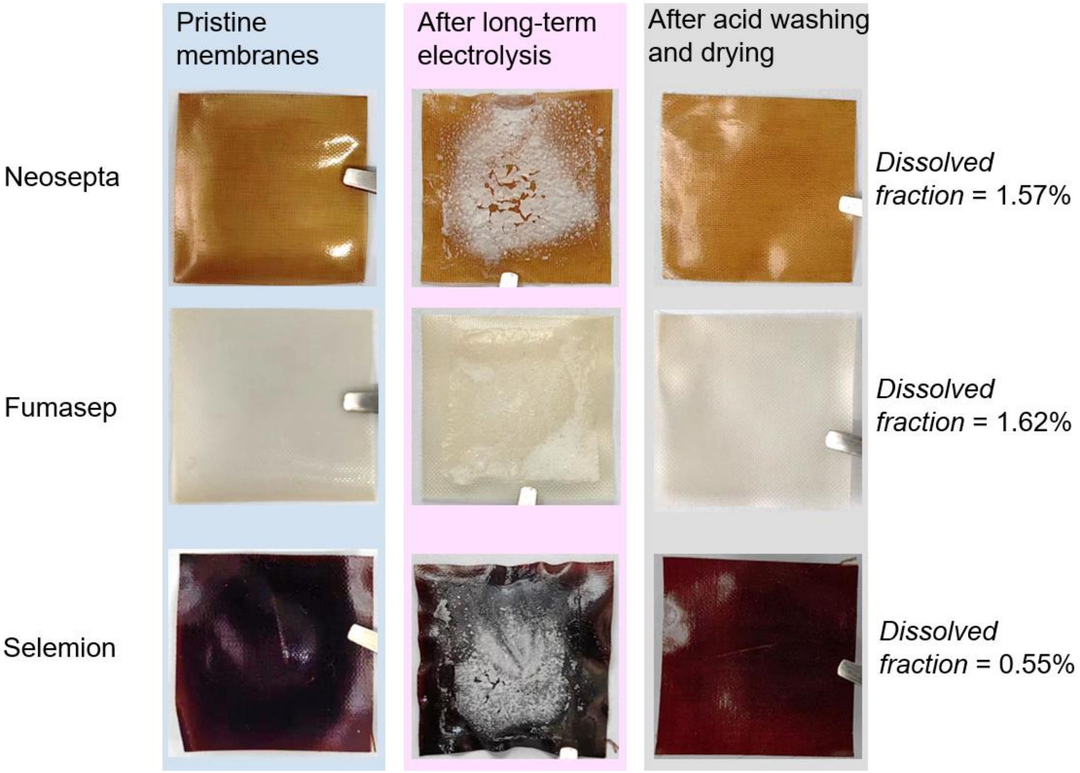

Figure S18. The images of the Neosepta, Fumasep, and Selemion CEMs at the initial stage, after the long-term electrolysis (1.0 A for 100 h), and washed by acid solution, as well as their calculated dissolved fraction after the electrolysis.

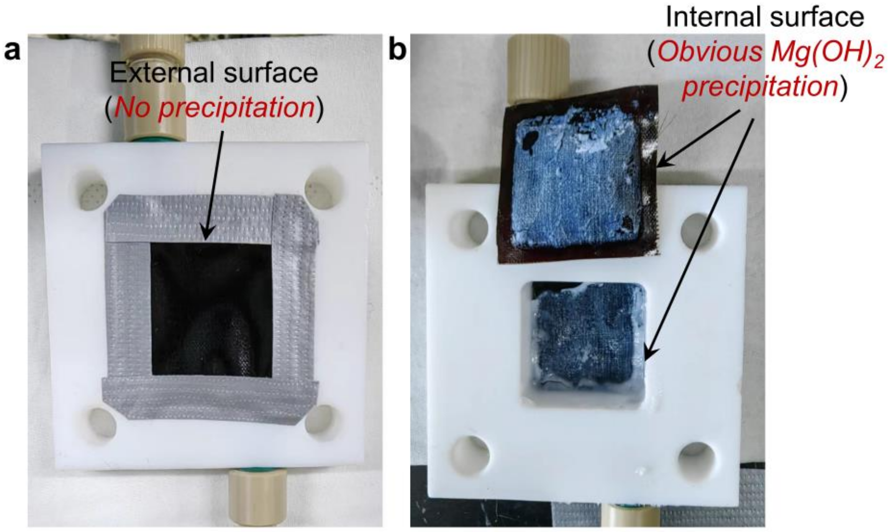

Figure S19. a, **b**. Images for the external (**a**) and internal surfaces (**b**) of Selemion CEMs after the potentiostatic electrolysis at 4.0 V for 1 h, respectively.

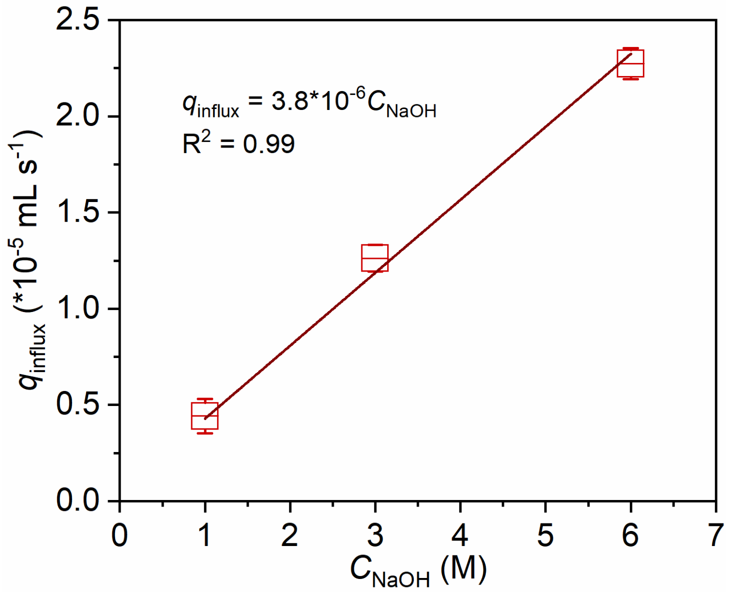

Figure S20. Function of q_{influx} *versus* C_{NaOH} in the system with seawater as the water source. The corresponding fitting curve of q_{influx} and C_{NaOH} exhibits a good linear relationship with the equation of $q_{influx} = 3.8*10^{-6}C_{NaOH}$ ($R^2 = 0.99$). Also, the D value in this system can be calculated as $4.8*10^{-6}$ based on the known surface area of the adopted membrane (0.785 cm^2). Thus, the relation between q_{influx} and C_{NaOH} can be described as $q_{influx} = 4.8*10^{-6}SC_{NaOH}$. According to this, the conditions for a continuous DSS system ($q_{influx} = q_{outflux}$) involving i and C_{NaOH} can be obtained ($i = 0.051SC_{NaOH}$).

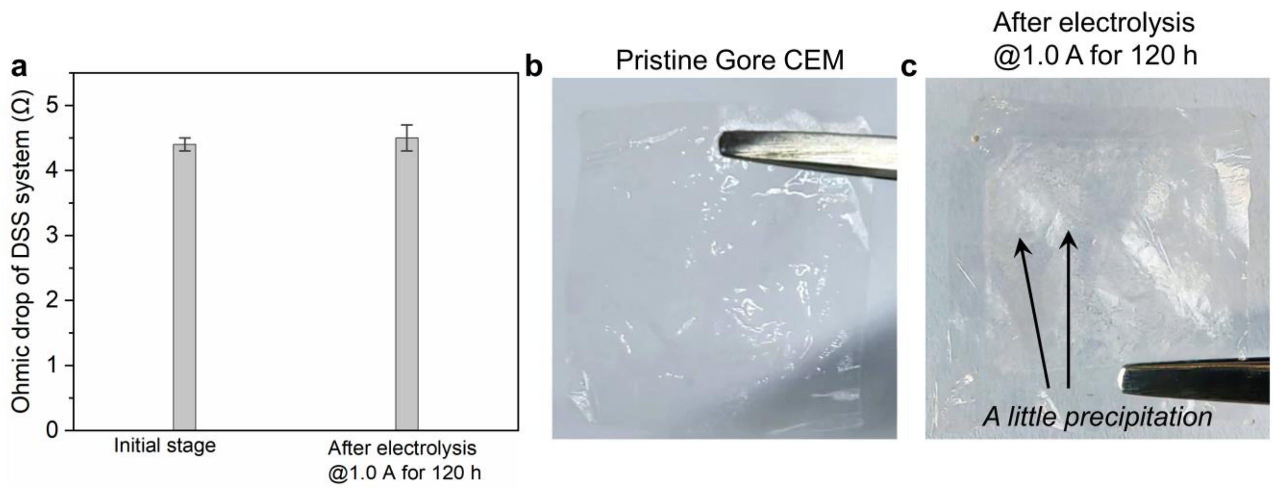

Figure S21. **a**, Ohmic drops of the DSS system at the initial stage and after the electrolysis at 1.0 A for 120 h. **b**, **c**, The pristine Gore CEM (**b**) and the Gore CEM after the electrolysis at 1.0 A for 120 h (**c**). As shown in Figure S20c, a little precipitation can be observed on the surface of the membrane.

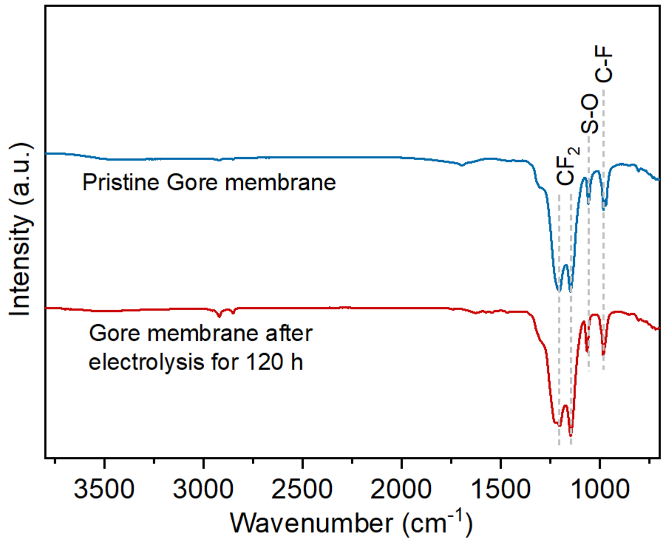

Figure S22. The attenuated total reflection (ATR) spectra of the Gore membrane before and after the electrolysis.

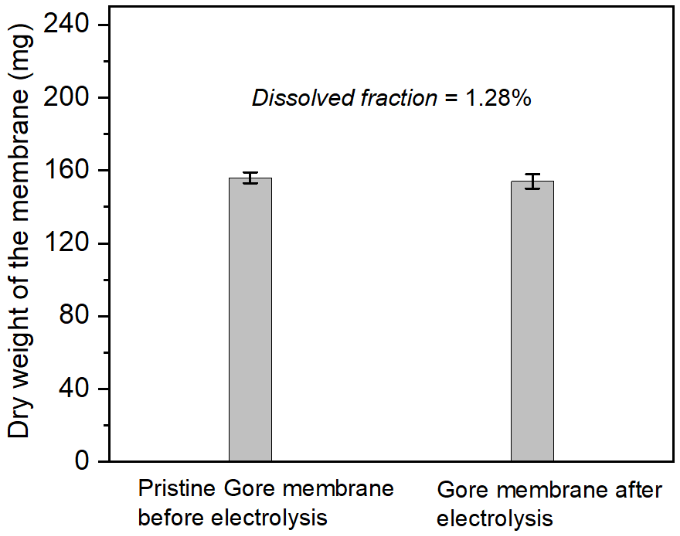

Figure S23. The dry weight of the Gore membrane before and after the electrolysis as well as the calculated dissolved fraction after the electrolysis.

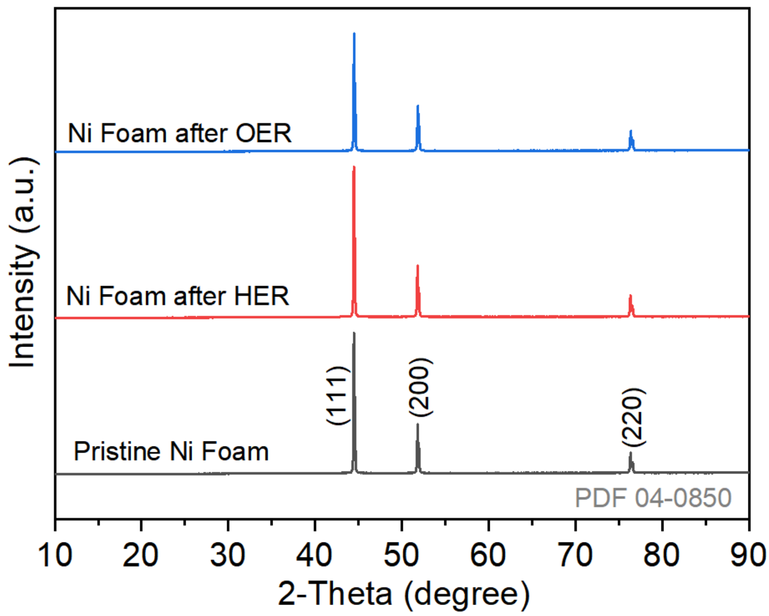

Figure S24. XRD patterns of pristine Ni foam and the Ni foams used as cathode (HER) and anode (OER) after the DSS in natural seawater at 1.0 A for 120 h.

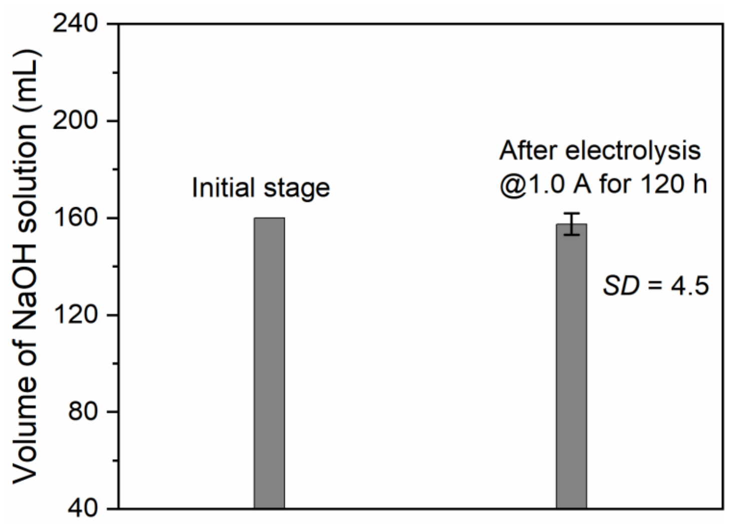

Figure S25. The volume of NaOH solution (2.9 M) before and after the continuous DSS at 1 A for 120 h. The test is conducted three times, and the corresponding standard error (*SD*) is calculated to be 4.5 mL.

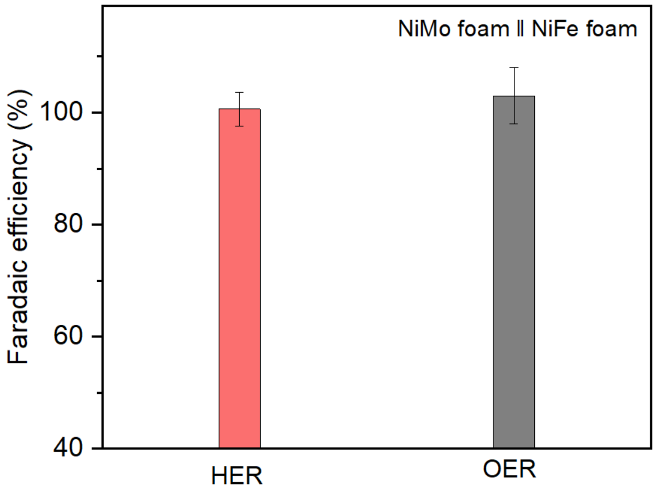

Figure S26. HER/OER FEs of the DSS system coupled with NiMo and NiFe foams under galvanostatic electrolysis at 1.0 A. It can be found that, the NiMo foam||NiFe foam system can also render ~100% Faradaic efficiencies for HER and OER, further demonstrating the practical superiority of our electrolyzer design concept.

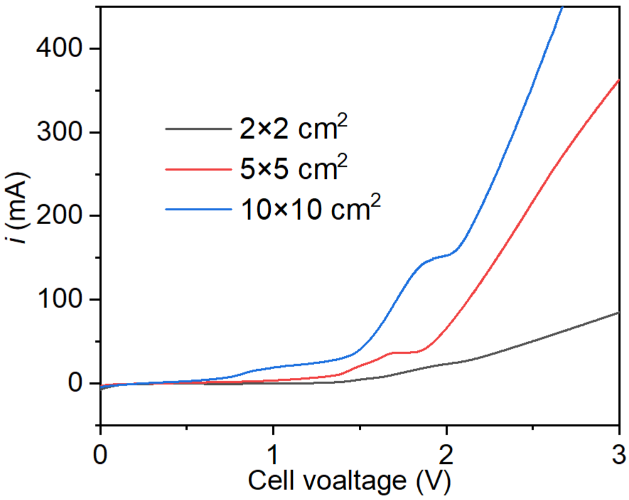

Figure S27. LSV curves of the scaled-up electrolyzers with various membrane sizes of 2 × 2, 5 × 5, and 10 × 10 cm^2, respectively (NaOH concentration: 1.0 M, CEM: Gore).

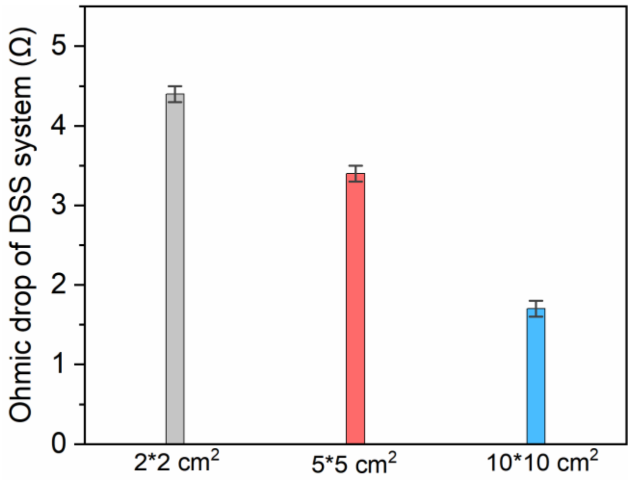

Figure S28. The ohmic drop of the system with different membrane sizes of 2 × 2, 5 × 5, and 10 × 10 cm^2. The tests were conducted with seawater and 1.0 M NaOH solution.

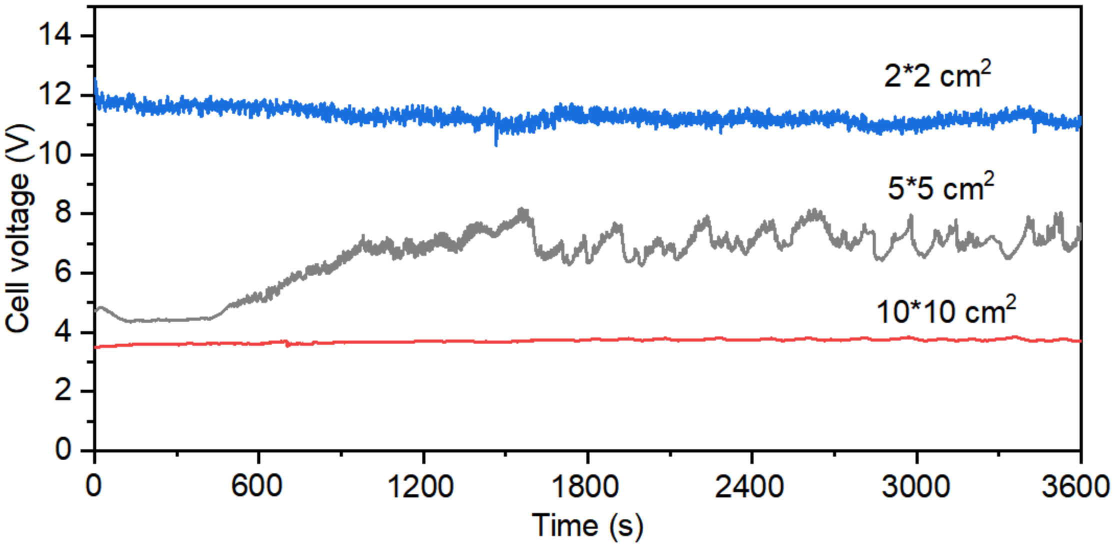

Figure S29. Galvanostatic electrolysis curves of the scaled-up DSS system with various membrane sizes of 2 × 2, 5 × 5, and 10 × 10 cm^2 at 1.0 A, respectively.

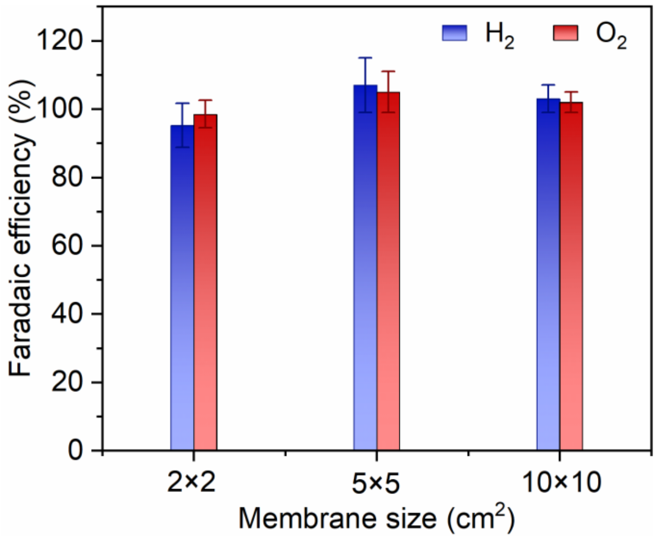

Figure S30. HER/OER FEs of scaled-up DSS systems under galvanostatic electrolysis at 1.0 A for 1 h.

Supplementary Tables

Table S1. Properties of the adopted commercial CEMs.

Type of CEM	Thickness (μm)	Area resistance (Ω cm^2)	Ion transport capacity (meq g^{-1})	Burst strength (MPa)
GORE-SELECT® Gore M788.12[a]	12	0.12	−	70
ASTOM Neosepta® CIMS[b]	150	1.8	0.98	>0.1
FUMA Fumasep FKB-PK-130[a]	130	4−6	0.8−1.0	40−80
AGC Selemion® CSO[b]	100	5	−	0.9

[a]The data were provided by the product brochures from the manufacturers.
[b]The data were collected by the product brochures from the manufacturers as well as the reported work[1-3].

Table S2. Concentration of the major constituents in seawater employed in this work.

Species	Concentration (mg L^{-1})
Cl^-	12715
SO_4^{2-}	1464
Na^+	9823
Mg^{2+}	125
Ca^{2+}	10
K^+	254

Table S3. Comparison of H_2 production performance of our DSS system with the state-of-art electrolysis systems in seawater.

Catalyst	Current (mA)	Geometric area of the electrode (cm^2)	Current density (mA cm^{-2})	Cell voltage (V)	Energy consumption (kWh Nm^{-3} H_2)	Ref.
NiMo foam||NiFe foam	1000	4.0	250	~2.7	~6.2	***This work***
PtNi mesh||Mo-Ni_3S_2/Ni foam	NA	NA	250	2.3	~4.2	*Nature* **2022**, *612*, 673–678
Cr_2O_3–CoO_x||Cr_2O_3–CoO_x	NA	NA	500	~2.2	NA	*Nat. Energy* **2023**, *8*, 264–272
Karst Ni foam||Karst Ni foam	2.5	0.25	10	1.79	NA	*Energy Environ. Sci.* **2020**, *13*, 174–182
Pt mech||Pt mech	250	NA	NA	NA	NA	*Proc. Natl. Acad. Sci. U. S. A.* **2021**, *118*, e2024855118
Ni-mesh||stainless-steel mesh	125	5.0	25	NA	NA	*ACS Appl. Energy Mater.* **2022**, *5*, 1403–140

References

1. Zhang, W.; Miao, M.; Pan, J.; Sotto, A.; Shen, J.; Gao, C.; der Bruggen, B. V., Separation of divalent ions from seawater concentrate to enhance the purity of coarse salt by electrodialysis with monovalent-selective membranes. *Desalination* **2017,** *411*, 28−37.
2. Pang, X.; Tao, Y.; Xu, Y.; Pan, J.; Shen, J.; Gao, C., Enhanced monovalent selectivity of cation exchange membranes via adjustable charge density on functional layers. *J. Membrane Sci.* **2020,** *595*, 117544.
3. Sugimoto, Y.; Ujike, R.; Higa, M.; Kakihana, Y.; Higa, M., Power generation performance of reverse electrodialysis (RED) using various ion exchange membranes and power output prediction for a large RED stack. *Membranes* **2022,** *12* (11), 1141.
